# The global distribution and transmission limits of lymphatic filariasis: past and present

**DOI:** 10.1186/s13071-014-0466-x

**Published:** 2014-10-11

**Authors:** Jorge Cano, Maria P Rebollo, Nick Golding, Rachel L Pullan, Thomas Crellen, Anna Soler, Louise A Kelly- Hope, Steve W Lindsay, Simon I Hay, Moses J Bockarie, Simon J Brooker

**Affiliations:** Faculty of Infectious and Tropical Diseases, London School of Hygiene & Tropical Medicine, London, United Kingdom; NTD Support Center, Task Force for Global Health, Emory University, Atlanta, United States of America; Department of Parasitology, Centre for Neglected Tropical Diseases, Liverpool School of Tropical Medicine, Liverpool, United Kingdom; Department of Zoology, Spatial Ecology and Epidemiology Group, University of Oxford, Tinbergen Building, South Parks Road, Oxford, UK; School of Biological and Biomedical Sciences, Durham University, Durham, United Kingdom

**Keywords:** Lymphatic filariasis, Global distribution, Transmission limits, Boosted regression tree modelling

## Abstract

**Background:**

Lymphatic filariasis (LF) is one of the neglected tropical diseases targeted for global elimination by 2020 and to guide elimination efforts countries have, in recent years, conducted extensive mapping surveys. Documenting the past and present distribution of LF and its environmental limits is important for a number of reasons. Here, we present an initiative to develop a global atlas of LF and present a new global map of the limits of LF transmission.

**Methods:**

We undertook a systematic search and assembly of prevalence data worldwide and used a suite of environmental and climatic data and boosted regression trees (BRT) modelling to map the transmission limits of LF.

**Results:**

Data were identified for 66 of the 72 countries currently endemic and for a further 17 countries where LF is no longer endemic. Our map highlights a restricted and highly heterogeneous distribution in sub-Saharan Africa, with transmission more widespread in West Africa compared to east, central and southern Africa where pockets of transmission occur. Contemporary transmission occurs across much of south and South-east Asia and the Pacific. Interestingly, the risk map reflects environmental conditions suitable for LF transmission across Central and South America, including the southern States of America, although active transmission is only known in a few isolated foci. In countries that have eliminated LF, our predictions of environmental suitability are consistent with historical distribution.

**Conclusions:**

The global distribution of LF is highly heterogeneous and geographically targeted and sustained control will be required to achieve elimination. This first global map can help evaluate the progress of interventions and guide surveillance activities.

**Electronic supplementary material:**

The online version of this article (doi:10.1186/s13071-014-0466-x) contains supplementary material, which is available to authorized users.

## Background

Lymphatic filariasis (LF) is a mosquito-borne disease which in its advanced forms can manifest as severe lymphoedema, hydrocele and elephantiasis [1]. The majority of global cases are caused by *Wuchereria bancrofti*, with *Brugia malayi* and *B. timori* as important local causes of the disease in South-east Asia. These nematode parasites are transmitted by various species of mosquito vectors from the genera *Anopheles*, *Aedes*, *Culex*, *Mansonia* and *Ochlerotatus*. LF is one of nine infectious diseases targeted for global elimination [2]. The selection of LF for elimination was based on (i) the absence of animal reservoirs for *W. bancrofti* (the most common form of LF) and only a small animal reservoir for *B. malayi* (which occurs in restricted foci) [3],[4], (ii) the existence of effective and practical interventions to interrupt transmission, and (iii) availability of an accurate diagnostic tool [5]. The main intervention strategy is mass drug administration (MDA) with albendazole in combination with diethylcarbamazine (DEC) (or ivermectin in countries where onchocerciasis is endemic) to entire communities in districts where the prevalence of LF is equal or more than 1% [6], supported by vector control to reduce exposure to mosquitoes and morbidity management to alleviate suffering and prevent disability of those affected by the disease [7],[8]. During the last half century, several countries have successfully eliminated LF, including Japan, China, South Korea, the Solomon Islands, Egypt and Togo [9]-[13].

A key component of national elimination programmes is a detailed understanding of the geographical distribution of LF so that all endemic areas can be targeted. Early attempts to map the distribution of LF included detailed literature reviews [14]-[20] or mapping at national or sub-national levels [21]-[26]. Although useful for estimating the global burden of LF [27], such national estimates belie the highly focal distribution of LF [28]-[32] and cannot be used to geographically target control. The mapping of LF has in recent years been greatly facilitated by the use of simple and rapid detection tests for *W. bancrofti* (antigen-based test) and *Brugia* spp (antibody-based test), based on the immuno-chromatographic test (ICT card test), which avoids the need to collect blood at night and the time-consuming preparation and examination of blood slides [33]-[38]. By the end of 2012, 59 out of the 72 endemic countries had completed national mapping surveys [39]. The results of these surveys highlight the marked within-country geographical heterogeneity [26],[40]-[46]. LF mapping is ongoing in the remaining endemic countries except Eritrea, where it has not started yet [2].

To augment available field surveys, a number of recent studies have sought to predict the distribution of LF on the basis of climatic and environmental factors. This approach is predicated on the fact that *W. bancrofti* is inefficiently transmitted, requiring thousands of infective bites to establish a patent infection [47], and as such LF is only likely to occur where climatic conditions are suitable to support mosquito vector populations over extended time periods [14],[48]. An early attempt to develop a risk map for LF in Africa was by Lindsay and Thomas in 2000, based on data from 32 studies using frequentist logistic regression and coarse-resolution environmental data [49]. More recently, Slater and Michael have used maximum entropy ecological niche modelling [50] and Bayesian model-based geostatistics [51] to predict the geographical occurrence and distribution of LF in Africa. Risk maps using environmental factors or spatial interpolation have also been developed at national or sub-national scales in West Africa [52], Egypt [53] and India [54].

Building on this previous work, we describe a new initiative to develop a global atlas of LF infection, which aims to collate all available survey data into a single, freely available resource and describe the historical and contemporary distribution of LF. Understanding these distributions has more than cartographic interest. First, changes in the epidemiology of LF over time can be quantified. Second, factors underlying such changes can be investigated in an effort to assess the degree to which changes are directly related to the scaling-up of interventions or other factors. Third, analysis of the historical risks of infection prior to large-scale intervention can be used to quantify the intervention needs required to reach programme goals [55], identify factors that contribute to the persistence of transmission, help define the intrinsic sensitivity (receptivity) of transmission to future changes in the intensity and frequency of control [56]-[59], and provide a basis to stratify surveillance activities. The specific aims of the present paper are (i) to detail the methods and approaches used to develop the database, (ii) to map historical and contemporary distributions of LF, and (iii) to delineate the global transmission limits of LF. The work is conducted within the context of the Global Atlas of Helminth Infections (www.thiswormyworld.org) [60] which aims to develop a suite of geographical resources and tools for neglected tropical diseases (NTDs).

## Methods

### Approaches to the diagnosis and mapping of LF

Night collection and examination of blood slides is considered the gold standard approach for detecting microfilariae. However, the sensitivity of this approach crucially relies on the volume of blood sampled [61]. Other parasitological methods, such as Knott’s concentration test and membrane filtration [62], increase the sensitivity of diagnosis but are prohibitively expensive for routine use. Alternatively, the presence of *W. bancrofti* antigenaemia can be detected using the ICT card test [33]-[35] and presence of specific IgG4 antibodies to *Brugia* spp can be detected using the Brugia Rapid™ test [36]-[38]. Surveys for the mapping of LF have been based on a variety of sampling designs, including the rapid geographical assessment of Bancroftian filariasis (RAGFIL) method [29],[63], lot quality assurance sampling [64],[65], population-based household surveys [66] and sentinel site surveys [43],[67],[68], with the choice of survey methodology dependent on available resources and the stage of the control programme [69].

### Identification of survey data

Our approach to identify suitable data follows that developed originally by Norman Stoll in his seminal work *This Wormy World* in 1947 [70], and adopted by efforts to map malaria [71],[72] as well as soil-transmitted helminth and schistosome infections [73]-[75]. Relevant data on the prevalence of LF were identified through a combination of (i) structured searches of electronic bibliographic databases, (ii) additional searches of the `grey’ literature, including unpublished surveys and government and international archives, and (iii) direct contact with researchers and control programme managers. The online databases PubMed, MEDLINE, EMBASE and SCOPUS were used to identify relevant studies for LF, using the following predefined Medical Subject Heading (MeSH) terms: lymphatic filariasis, bancroftian filariasis, *Wuchereria bancrofti*, *Brugia malayi*, *Brugia timori*, malayan filariasis AND current and former country names. All permutations of MeSH terms were entered, with no restrictions on language or date of publication. The abstracts of returned articles were reviewed and if they did not explicitly report prevalence data, they were discarded. When the abstract was not available, pre-selection was made according to the title. Authors were contacted when additional information was required or when data needed to be disaggregated. Studies were included if they provided: (i) the number of people surveyed, (ii) the number of LF positive cases, (iii) details about the methodology of diagnosis and (iv) details of the specific site where they were conducted, regardless of the administrative level. Surveys reporting only prevalence data without provision of the denominator were also included as these can be used to delineate the limits of transmission. Baseline data from clinical and diagnostic trials fulfilling the inclusion criteria were abstracted, whereas surveys carried out at hospitals, prisons, mental institutions or military facilities were excluded.

Data were extracted into a customized Microsoft Access (Microsoft 2007) database and linked to an identical SQL Server database. Abstracted data included three types of information: (i) epidemiological data on survey method, type of diagnostic method, dates, age range and gender of targeted people, time of survey (day or night), ongoing control activities and number of MDA rounds undertaken in the area at the time of survey, number of people sampled, number of positive individuals and prevalence, diagnostic method used, blood sampling volumes for detection of microfilaraemia and, where available, morbidity data based on hydrocele and/or lymphoedema; (ii) each record was assigned a unique identifier which linked the record to the source publication which was included in an Endnote library (Thomson Reuters 2010), with a pdf copy of each publication obtained; and (iii) all data were linked to first and second administrative units, based on the United Nations’ Second Administrative Level Boundaries (SALB) database [76] and the GADM version 2 database of Global Administrative Areas [77].

### Geo-positioning of survey data

**Figure 1:**
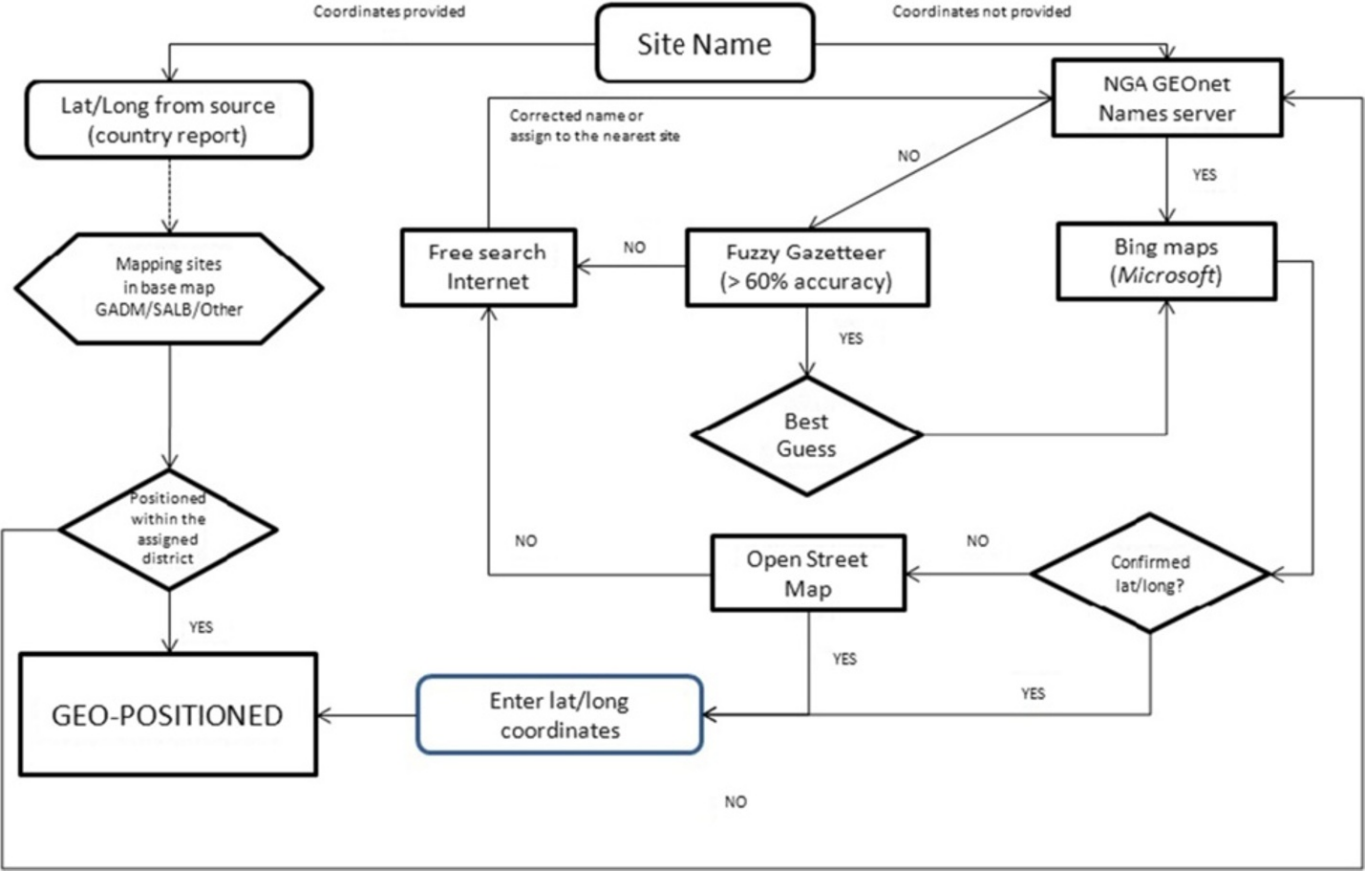
**Decision-based algorithm for the geopositioning of community surveys.** This algorithm was developed to ensure the maximum level of accuracy when geopositioning survey data. Briefly, when longitude and latitude of a survey site were provided by a publication, they were cross-checked against a range of cartographic resources, including NGA GEOnet Names server (http://earth-info.nga.mil/gns/html/), Bing maps (http://www.bing.com/maps/), Fuzzy Gazetteer/ISODP project (http://isodp.hof-university.de/fuzzyg/query/) and OpenStreetMap (http://www.openstreetmap.org/). The same resources were used to geoposition surveys for which coordinates were not provided.

A decision-based algorithm (Figure 1) was applied to determine the longitude and latitude of survey locations, using a variety of gazetteers: Bing Maps [78], GeoNet Names Server [79], Fuzzy gazetteers [80] and the Open Street Map project [81]. Geographical coordinates provided by source publications were cross-checked against these resources. The reliability of geopositioning was established on a scale of 0 to 4, `0’ being no coordinates found, `1’ highly reliable, `2’ fairly reliable (spelling differences in 1 or 2 characters), `3’ less reliable (spelling differences in various characters) and `4’ highly unreliable when less than 60% of name similarity in gazetteers or when located in nearby sites. All geographic coordinates were standardized to decimal degrees in order to be displayed in the WGS84 geographic coordinate system. Ideally, surveys were located to a point location but in certain instances surveys were located to a wide-area polygon (10-25 km^2^ area), and then the centroid of the polygon used.

### Environment and demographic covariates

Geolocated prevalence data were linked to a range of environmental and climatic variables which are known to affect the development and survival of LF parasites and its mosquito-vector species [82]-[86] (Table 1). These data included mean, minimum and maximum estimates of temperature and precipitation at 1 km^2^ resolution obtained from WorldClim database [87]. Elevation data at 1 km resolution were derived from gridded digital elevation models (DEM) produced by the Shuttle Radar Topography Mission (SRTM). An aridity index, which is a generalized function of precipitation, temperature, and/or potential evapotranspiration, was obtained at 1 km resolution from CGIAR-CSI [88]. Estimates of averaged enhanced vegetation index for period 1981 to 2006 were derived from imagery obtained from the Advanced Very High Resolution Radiometer (AVHRR) instrument onboard the NOAA satellites series, available from the data library of the International Research Institute for Climate and Society at Columbia University (IRI) [89]. Land cover data were obtained from the GlobCover project [90] which comprises 22 land cover classes, which we aggregated into seven major classes potentially relevant to the eco-epidemiology of LF: agricultural lands, forest areas, shrubland, grasslands and woodlands, bare soil, urban areas, snow/ice and water areas.

**Table 1:** **Description of environmental variables used to model the global distribution of lymphatic filariasis transmission**

Environmental variable	Description
Mean minimum temperature	Mean of average monthly minimum temperature across all 12 months (period 1950-2000)
Annual mean temperature	Mean of average monthly mean temperature across all 12 months (period 1950-2000)
Precipitation in wettest quarter	Total precipitation in the wettest quarter
Precipitation in driest quarter	Total precipitation in the driest quarter
Annual precipitation	Mean of accumulative precipitation across all 12 months (period 1950-2000)
Aridity Index	Indicator of the degree of dryness of the climate at a given location, and result from dividing the mean annual precipitation by the mean annual potential evapo-transpiration
Elevation	Metres above the sea level obtained by radar imagery
EVI	Mean of 8-day composite MODIS images for the period 2001 to 2005. EVI raw values (scale from 0 to 1) have been multiplied by 1,000 for being storage as integers.
Global Land Cover 2006	A simplified version of seven major land cover classes obtained from the UN Global Land Cover (22 classes)
Population density (1995-2015)	Average population density 1995-2015
WHO regions	WHO regions: AFRO/EMRO (African and Eastern Mediterranean regions), AMRO (Americas region), SEARO (South-east Asia) and WPRO (western Pacific)
Accessibility	Cost (in time units) to access to large urban areas

Estimates of population density were obtained from the Gridded Population of the World (GPW) [91], which was used to classify areas as urban, peri-urban or rural areas, based on the assumption that urban extents (UE) have a population densities ≥1,000 persons/km^2^, peri-urban >250 persons/km^2^ within a 15 km distance from UE edge, and rural <250 persons/km^2^ and/or >15 km from the UE edge [92]. A gridded map of urban accessibility 1 km resolution was obtained from the European Commission Joint Research Centre Global Environment Monitoring Unit (JRC) [93]. This dataset defined urban accessibility as the predicted time taken to travel from that grid cell to a city of ≥50,000 persons in the year 2000 using land- or water-based travel. Finally, a gridded map at 5 km resolution of the main geographic regions (Europe, Asia, Africa and America) was created. Western Pacific countries were grouped upon the Asian region whilst countries located in the Arabian Peninsula were included within the African region.

To bring the spatial resolution of these covariate layers in line with the spatial accuracy of the survey data, covariates were resampled to a common 5 km resolution raster layout based on the WGS-1984 Web Mercator projection using ArcGIS 10.1. (ESRI Inc., Redlands CA, USA). Bilinear interpolation was applied to resample numeric (continuous) raster data sets, whereas nearest neighbour interpolation was used with ordinal raster layers. Possible collinearity between the covariates was explored using cross-correlations. All correlation coefficients were less than 0.7, indicating that covariates were largely orthogonal.

### Predicting the probability of LF transmission using boosted regression trees

Boosted regression trees (BRT) modelling was used for mapping the spatial limits of LF transmission. This approach has been shown to have higher predictive accuracy than other distribution models [94] and has been successfully applied to map dengue [95] and malaria vector mosquitoes [96]. BRT combines two machine learning approaches; regression trees (simple hierarchical models which allow non-linear effects of predictors) and boosting (fitting ensemble models by iterative improvements on the existing ensemble [97],[98]). A first step in the BRT approach is the definition of occurrence and absence data. Records of disease occurrence were defined as surveys during which one or more cases of LF were detected, regardless of diagnostic method used. Absence records were defined as surveys conducted prior to large-scale control activities and from which no cases of LF were reported. Because relatively few absence records were available (prevalence surveys are typically carried out in areas where disease presence is expected), these data were supplemented with pseudo-absence records following a similar procedure to that used for mapping dengue and malaria vectors [93],[94]. Five thousand pseudo-absence data points were generated at random in areas known not to be endemic for LF based on expert knowledge [14]-[20],[99] or those areas considered as unsuitable habitats for mosquito breeding - areas of bare and hyper-arid land, as classified by the GlobCover [90] and Global Aridity Index datasets [88]. In order to maximise the ability of the model to discriminate between suitable and unsuitable areas, regression weights were used to down-weight pseudo-absence records, so that the summed weights of the absence and pseudo-absence records matched that of the presence records.

In the second stage, eight environmental variables along with altitude, population density, accessibility and macro-geographical regions were used to predict LF occurrence in a single BRT model, in order to explore the relative importance of each factor in explaining the global occurrence of LF (Table 1). Those factors that contributed little (relative contribution <2%) to the single BRT model were disregarded to build the final ensemble BRT model. Thus, six covariates (precipitation in the wettest quarter, annual minimum temperature, population density 1990-2015, elevation, enhanced vegetation index and regions) were subsequently selected and eventually used to build the final risk map.

In order to estimate uncertainty in the model and the resulting risk maps, we finally fitted an ensemble of 120 BRT submodels, each fitted to a random bootstrap of the full dataset. The predicted distributions of LF from each of these submodels were then averaged to generate the final risk map. Predictive performance of each submodel was evaluated using the following statistics: proportion correctly classified (PCC), sensitivity (proportion of presences correctly classified), specificity (proportion of absences correctly classified), Kappa (*k*) and area under the receiver operator characteristic (AUC). The mean and confidence intervals for each statistic were used to evaluate the predictive performance of the ensemble BRT model. Marginal effect curves were plotted to visualise dependencies between the probability of LF occurrence and each of the covariates. These show the marginal effect of each covariate on the response after averaging the effects of all other covariates. Finally, the relative contribution of each covariate (the percentage of tree branches in each submodel that used the covariate) to the final BRT model was also quantified.

### Defining limits of transmission

The resulting predictive map quantifies the environmental suitability for LF transmission. In order to convert this continuous metric into a binary map outlining the limits of transmission, a threshold value of suitability was determined, above which transmission was assumed to be possible. Based on the receiver operating characteristic curve, a threshold value of environmental suitability was chosen which maximised the trade-off between sensitivity, specificity and PCC. Whilst the resulting map delineates environmental suitability for LF transmission, it may include areas where transmission does not actually occur, due either to the disease never having been imported into the area or the consequence of control leading to local elimination. To reflect this, we masked the environmental distribution map to remove areas which are known to be currently non-endemic according to WHO [39],[100],[101] and other sources [20],[40],[41],[44],[99]. Non-endemicity was considered when no cases had been reported for the last 10 years and transmission assessment surveys confirmed the interruption of LF transmission.

## Results

### LF database

The search strategy identified 9,033 surveys, conducted between 1902 and 2013 in 85 countries, which were eligible for inclusion. Summary characteristics of included surveys are reported in Table 2. An extended version of this table is additionally provided (see Additional file 1). Data were identified for 35 of 37 current endemic countries of sub-Saharan Africa and Eastern Mediterranean WHO-region (AFRO/EMRO); 9/9 from South East Asia region (SEARO); 18/22 from western Pacific region (WPRO); 4/4 from the Americas region (AMRO). Data were also available for a further 17 countries^a^ where LF is no longer endemic or where transmission has recently been declared as interrupted. Current endemic countries for which no data were available were Angola and Gabon, in the AFRO region, and Cambodia, Lao People’s Democratic Republic, Niue, and North Korea, in the SEARO and WPRO regions.

**Table 2:** **Characteristics of surveys included in the lymphatic filariasis database**

WHO - regions^&^	AFRO/EMRO	SEARO	WPRO	AMRO	Total
	N (%)	N (%)	N (%)	N (%)	N (%)
Current endemic countries	37	9	22	4	72
Countries with data	35	9	25	16	85
Number of surveys identified	5,468	1,764	1,322	479	9,033
Non-community data	673 (12.3)	246 (13.9)	201 (15.2)	43 (9.0)	1,163 (12.9)
Geopositioned	4,624 (84.6)	1,320 (74.8)	1058 (80.0)	418 (87.3)	7,420 (82.1)
Not geopositioned	171 (3.1)	198 (11.2)	63 (4.8)	18 (3.8)	432 (5.0)
*Indicators concerning to community-level geopositioned surveys*					
**Surveys by period**					
before 1990	1,157 (25.0)	688 (52.1)	774 (73.2)	316 (75.6)	2,935 (39.6)
1990-2000	441 (9.5)	146 (11.1)	116 (11.0)	70 (16.7)	773 (10.4)
2000-2010	2,040 (44.1)	486 (36.8)	159 (15.0)	32 (7.7)	2,717 (36.6)
2010-now	937 (20.3)	-	9 (0.9)	-	946 (12.7)
Unknown	49 (1.1)	-	-	-	49 (0.7)
**Type of survey**					
Mapping/prevalence	3,875 (83.8)	924 (70.0)	954 (90.2)	400 (95.7)	6,153 (82.9)
SS/Spot check	749 (16.2)	280 (21.2)	94 (8.9)	18 (4.3)	1,141 (15.4)
TAS	-	6 (0.5)	10 (0.9)	-	16 (0.2)
Passive recording	-	110 (8.3)	-	-	110 (1.5)
**Diagnostic method**					
Clinical	24 (0.5)	119 (9.0)	5 (0.5)	1 (0.2)	149 (2.0)
Parasitological	1,939 (41.9)	1,086 (82.3)	892 (84.3)	370 (88.5)	4,287 (57.8)
Serological	2,423 (52.4)	69 (5.2.)	114 (10.8)	30 (7.2)	2,636 (35.5)
Other	238 (5.1)	46 (3.5)	47 (4.4)	17 (4.1)	348 (4.7)
**MDA Implemented**					
Unknown	19 (0.6)	36 (2.7)	-	18 (4.3)	84 (1.1)
Pre-intervention	3,918 (84.7)	827 (62.7)	558 (52.7)	312 (74.6)	5,615 (75.7)
Post-intervention	676 (14.6)	457 (34.6)	500 (47.3)	88 (21.1)	1,721 (23.2)

^&^AFRO - African Regional Office, AMRO - Americas Regional Office, EMRO - Eastern Mediterranean Regional Office, SEARO - South East Asian Regional Office, WPRO - Western Pacific Regional Office.

Of eligible surveys, 7,852 (87.1%) represented disaggregated, community-based surveys of which 7,420 (94.3%) could be geo-positioned: 6,442 to point locations and 978 to small areas, such as households with scattered distributions, small islands and small administrative areas. Data extracted from published sources accounted for 53.2% of included survey locations and were the main source of information in the SEARO and WPRO regions. Grey literature, which included country reports, GAELF reports and other unpublished articles, accounted for 46.8% of survey locations, and were the main source of data in the AFRO/EMRO region. The majority (82.9%) of data points were obtained through mapping or prevalence surveys, whereas 1,141 (15.4%) were sentinel site surveys and 16 transmission assessment surveys. Among mapping/prevalence surveys, 609 (9.9%) surveys were obtained by lot quality assurance sampling (LQAS), mostly conducted after 2000. Finally, 110 surveys derive from a countrywide clinical survey in Thailand, 1949-1950, where lymphoedema of lower limbs were recorded upon systematic population screening by headmen of cantons [102] - this survey was included as it provided the best nationwide data on occurrence for Thailand.

**Figure 2:**
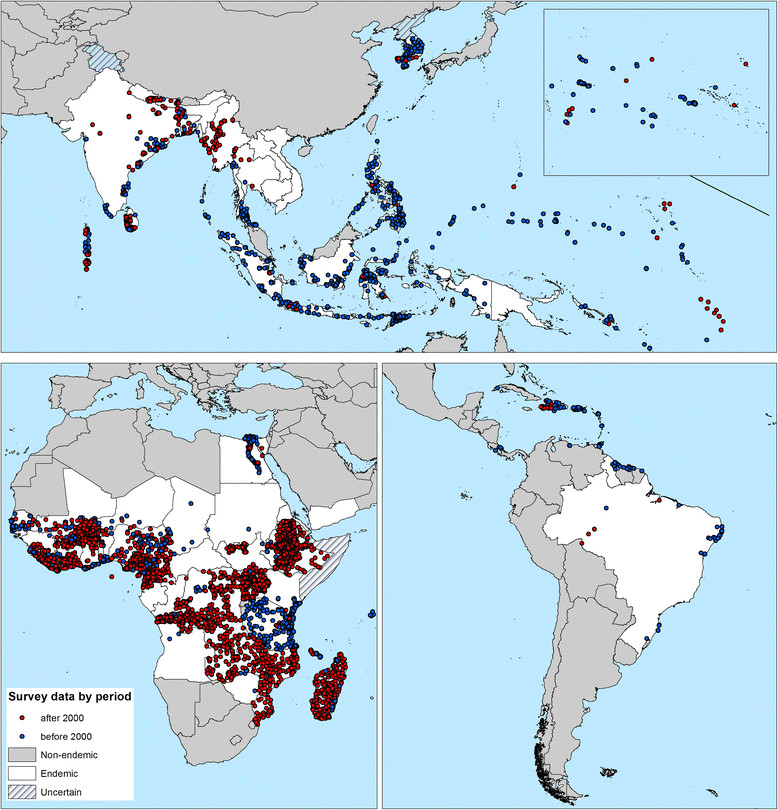
**The global distribution of data points included in the Global Atlas of Lymphatic Filariasis database by period of time.** Red = surveys undertaken post- 2000 when GFLEP was launched, and blue = before 2000. Current endemic countries are displayed in white, non-endemic countries in grey and hatching depicts countries where endemicity is uncertain.

**Figure 3:**
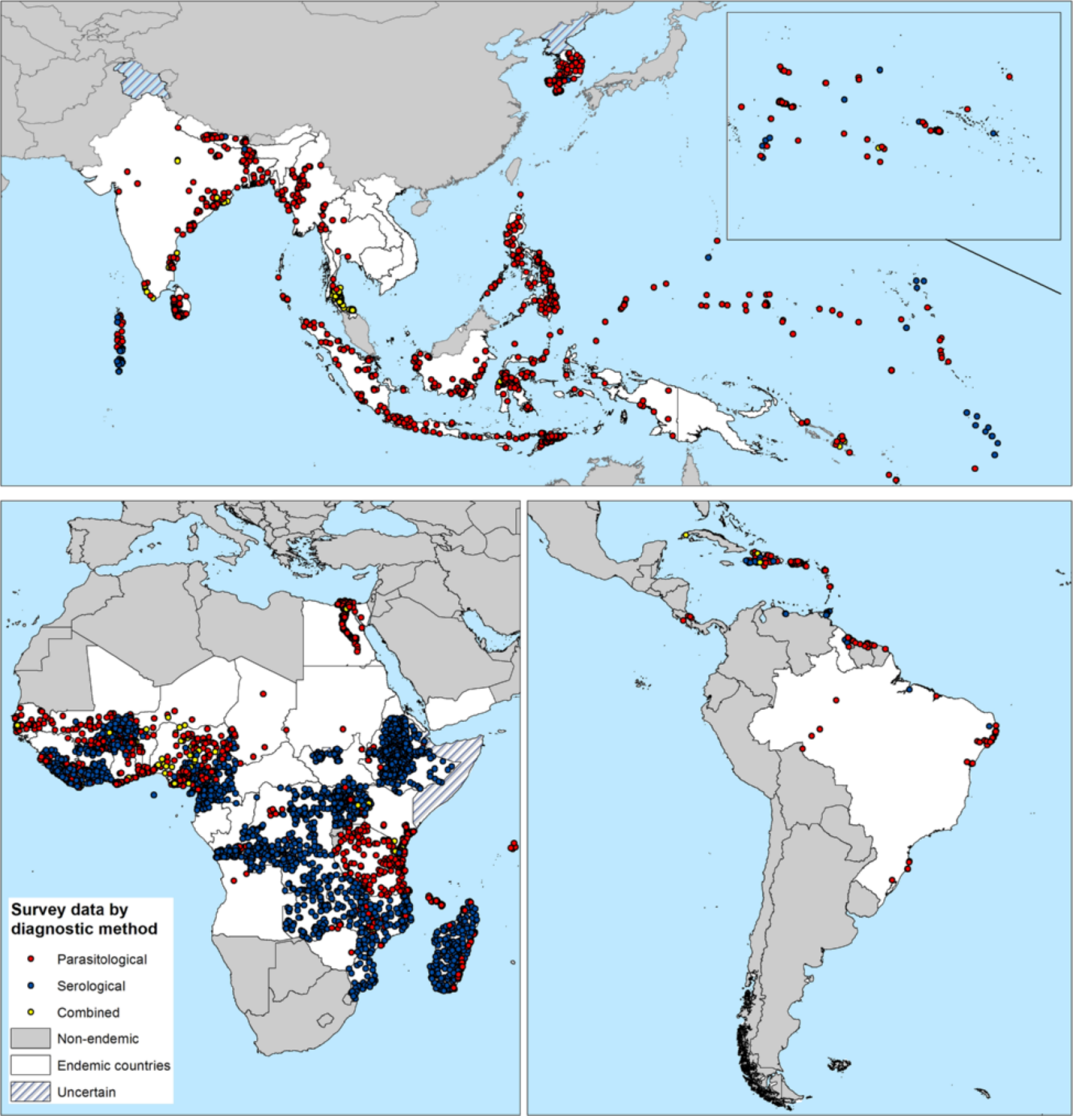
**The global distribution of data points included in the Global Atlas Lymphatic Filariasis database by diagnostic method.** Red = parasitological methods; blue = serological methods; and yellow = combination of methods.

Figures 2 and 3 show the geographical distribution of survey locations by time period and by diagnostic method, respectively. The date of surveys varied between regions. The majority (71.9%) of data gathered for the AFRO/EMRO region were collected post-2000, whilst for other regions much of the available data were collected pre-2000. Parasitological-based diagnosis accounted for 92.04% of surveys before 2000, whereas 63.8% of the 4,065 surveys undertaken after 2000 used serological tests. Only 348 (4.7%) surveys used two or more diagnostic methods.

### Factors associated with LF transmission

**Figure 4:**
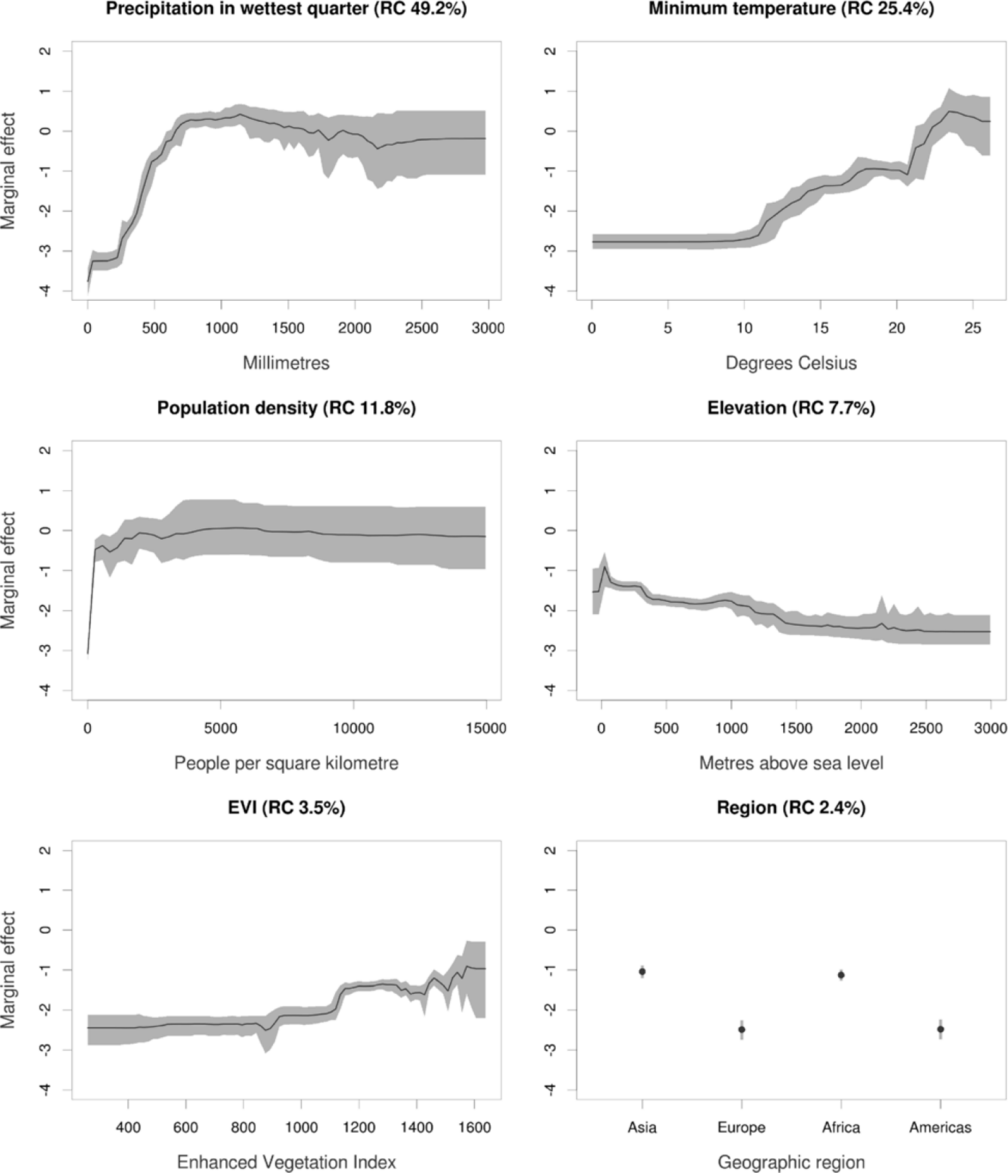
**Marginal effect curves for each covariate used in the ensemble of 120 boosted regression tree (BRT) models.** Black lines represent the mean marginal effect over all 120 BRT ensembles and grey envelopes the 95% bootstrap confidence interval. The *y-* axis is the untransformed logit response and *x-* axis is the full range of covariates values. The percentage values in parenthesis show the mean relative contribution of the covariate over all 120 sub-models of the ensemble.

A subset of 6,562 surveys (4,933 occurrences and 1,629 absences) available in the assembled LF database along with the generated pseudo-absence data (5,000) were used to model the global distribution of LF. Figure 4 shows the marginal effect of each variable on the response, averaging across the effects of all other variables, and its relative contribution to the final BRT model. High suitability for LF is positively associated with precipitation in the wettest quarter (reaching a plateau at rainfall greater than 1000 mm), increased vegetation cover, population density and minimum temperature (increasing from a minimum value of 10°C), and negatively associated with increasing elevation (Figure 4).

### Global LF transmission map

**Figure 5:**
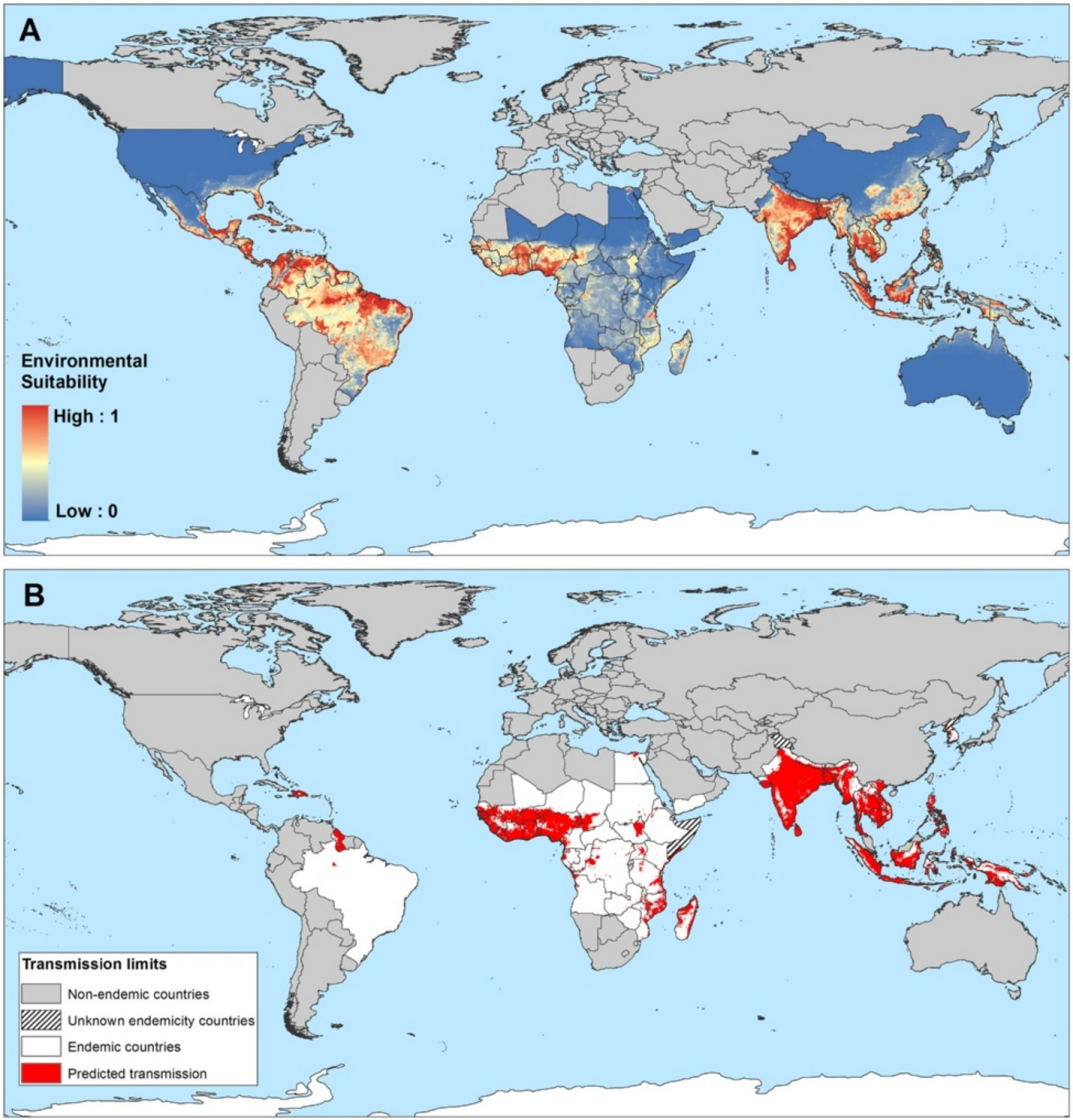
**Global environmental suitability (A) and limits (B) of lymphatic filariasis transmission as predicted by the final boosted regression trees model.** Countries that have never reported LF endemic infections are masked in grey, and areas suitable for LF transmission, as predicted by the BRT model, are displayed in red **(B)**.

**Figure 6:**
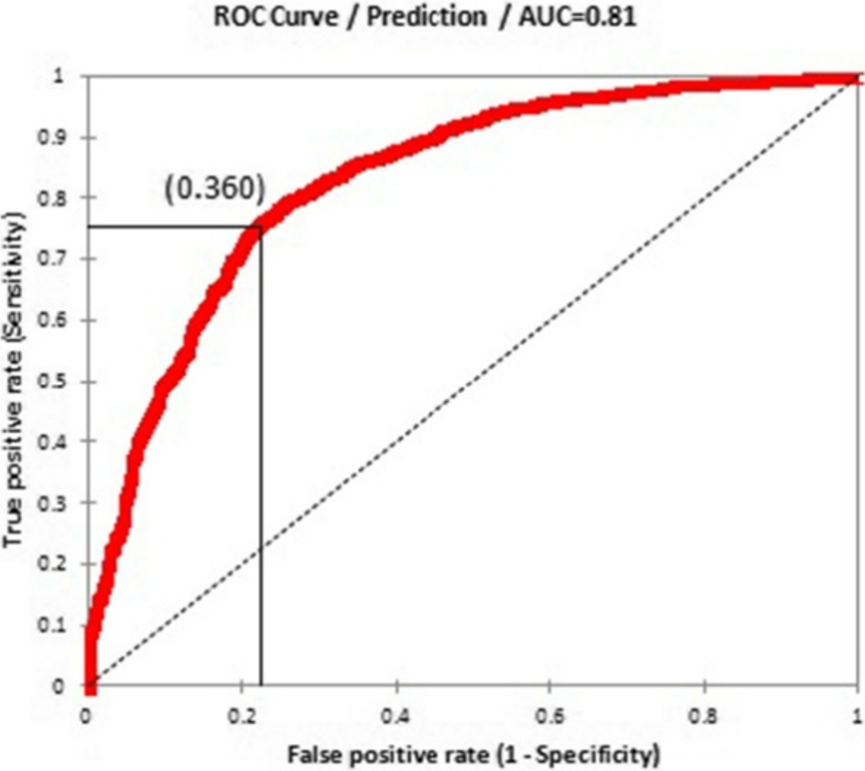
**Receiver Operating Characteristics curve for the occurrence of LF transmission and associated model validation statistics: AUC = 0.81 (95** **%** **CI: 0.78 – 0.83; sd: 0.01), sensitivity = 0.73 (95** **%** **CI: 0.64 – 0.79; sd: 0.02), specificity = 0.76 (95** **%** **CI: 0.7 – 0.83; sd: 0.02), proportion correctly classified (PCC) = 0.75 (95** **%** **CI: 0.72 – 0.78; sd: 0.01) and Kappa = 0.5 (95** **%** **CI: 0.44 – 0.56; sd: 0.02).** The environmental suitability threshold which provided the best trade-off between sensitivity, specificity and proportion of correctly classified was 0.360.

Figure 5A presents the global map of environmental suitability for LF transmission and suggests that this suitability occurs primarily in tropical and sub-tropical regions, with the highest suitability in parts of Central and Latin America, West Africa, coastal east and southern Africa, India, Southeast Asia, Indonesia, Papua New Guinea and western Pacific. Validation statistics indicated high predictive performance of the BRT ensemble model with area under the receiver operating characteristic (AUC) of 0.81 (95% CI: 0.78 – 0.83; sd: 0.01). An environmental suitability threshold of 0.36 provided the best discrimination between presence and absence records (Figure 6) and this threshold value was used to classify the environmental suitability map into a binary map of the environmental limits of transmission. This map and the map of the current transmission limits (excluding areas known to be non-endemic) are shown in Figure 5.

### LF transmission by region

**Figure 7:**
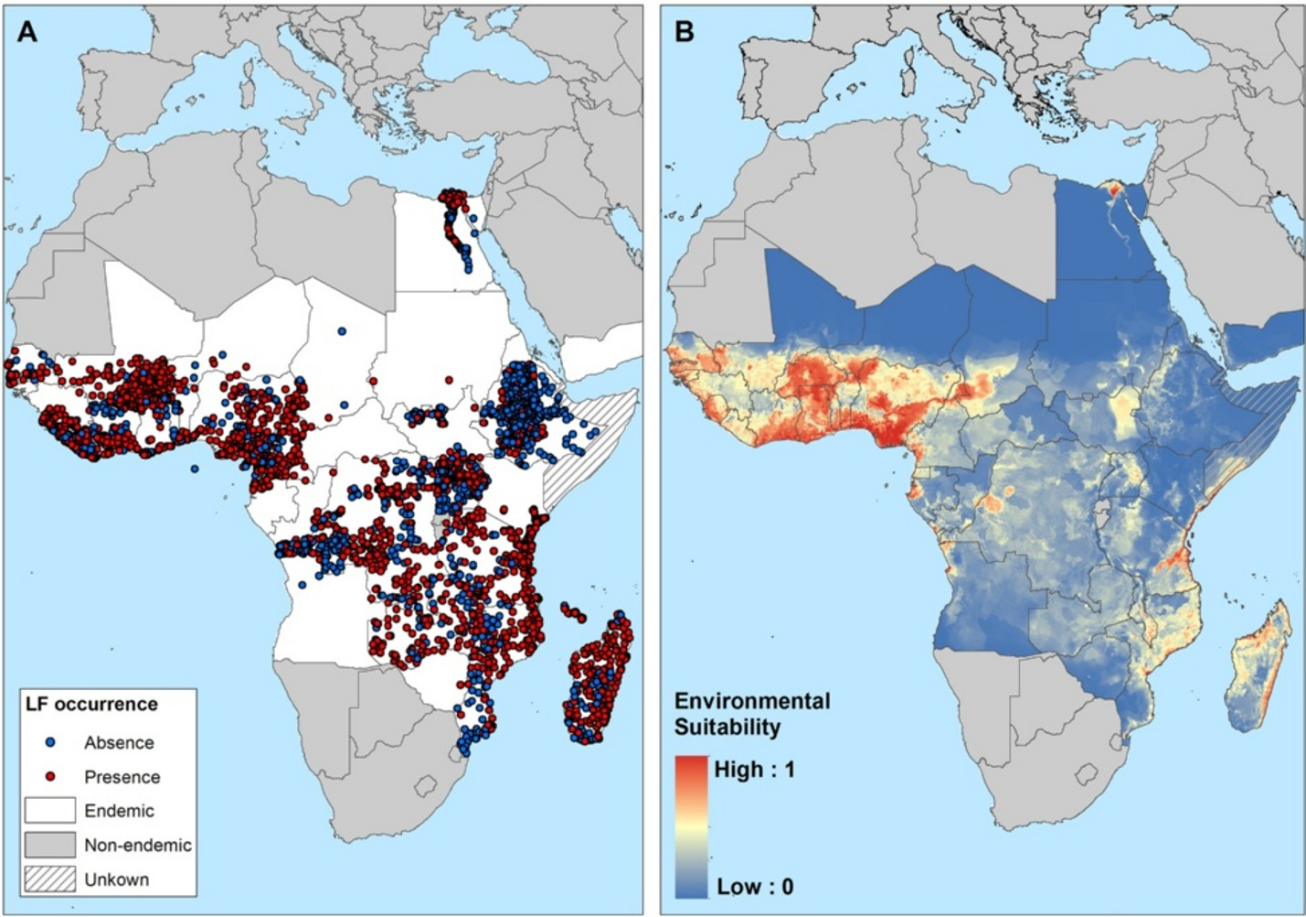
**Reported and predicted distribution of lymphatic filariasis in Africa. (A)** Observed occurrence and absence of LF and **(B)** environmental suitability for lymphatic filariasis transmission, as predicted by the final boosted regression trees model, in Africa.

**Figure 8:**
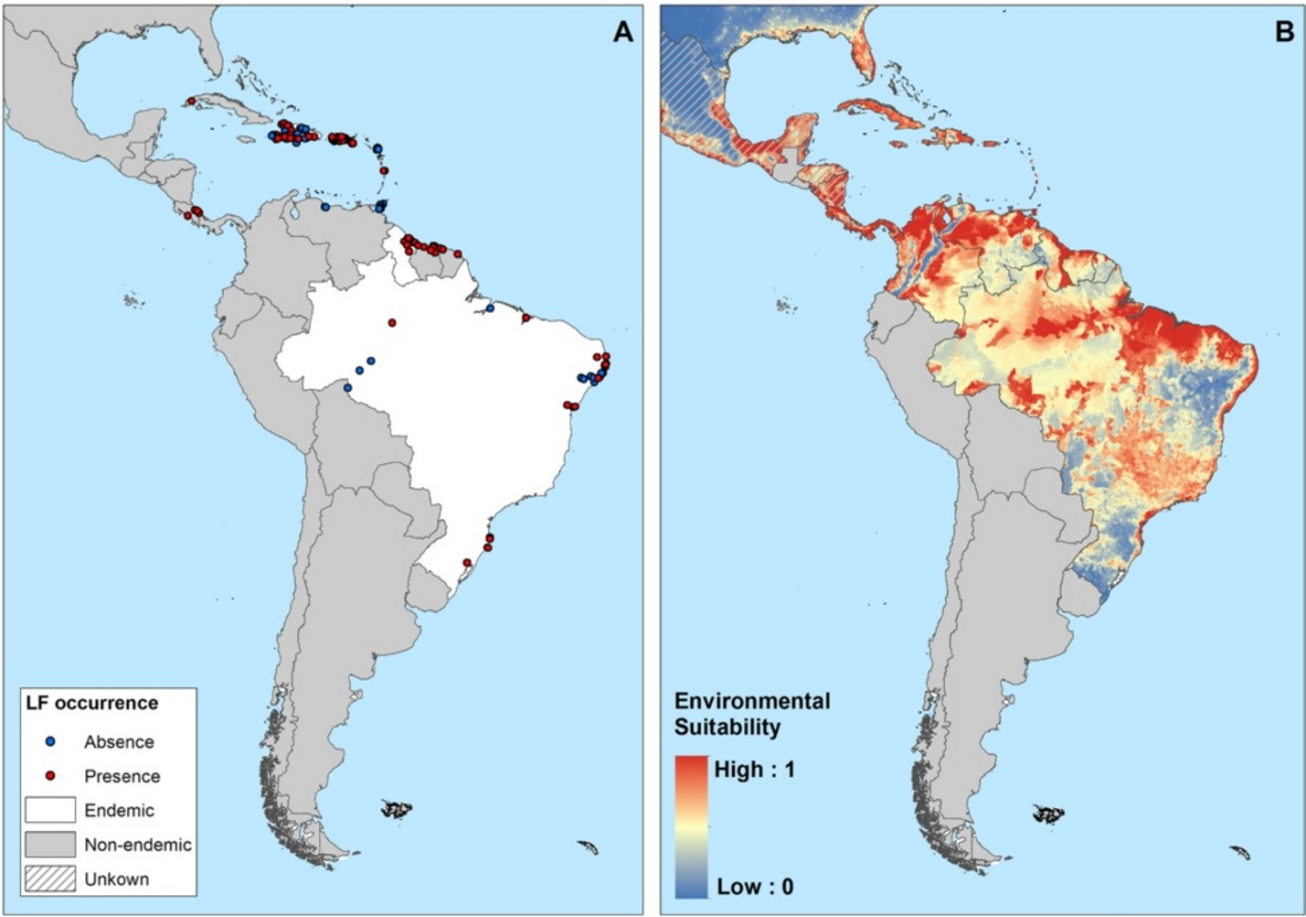
**Reported and predicted distribution of lymphatic filariasis in the Americas. (A)** Observed occurrence and absence of LF and **(B)** environmental suitability for lymphatic filariasis transmission, as predicted by the final boosted regression trees model, in the Americas.

**Figure 9:**
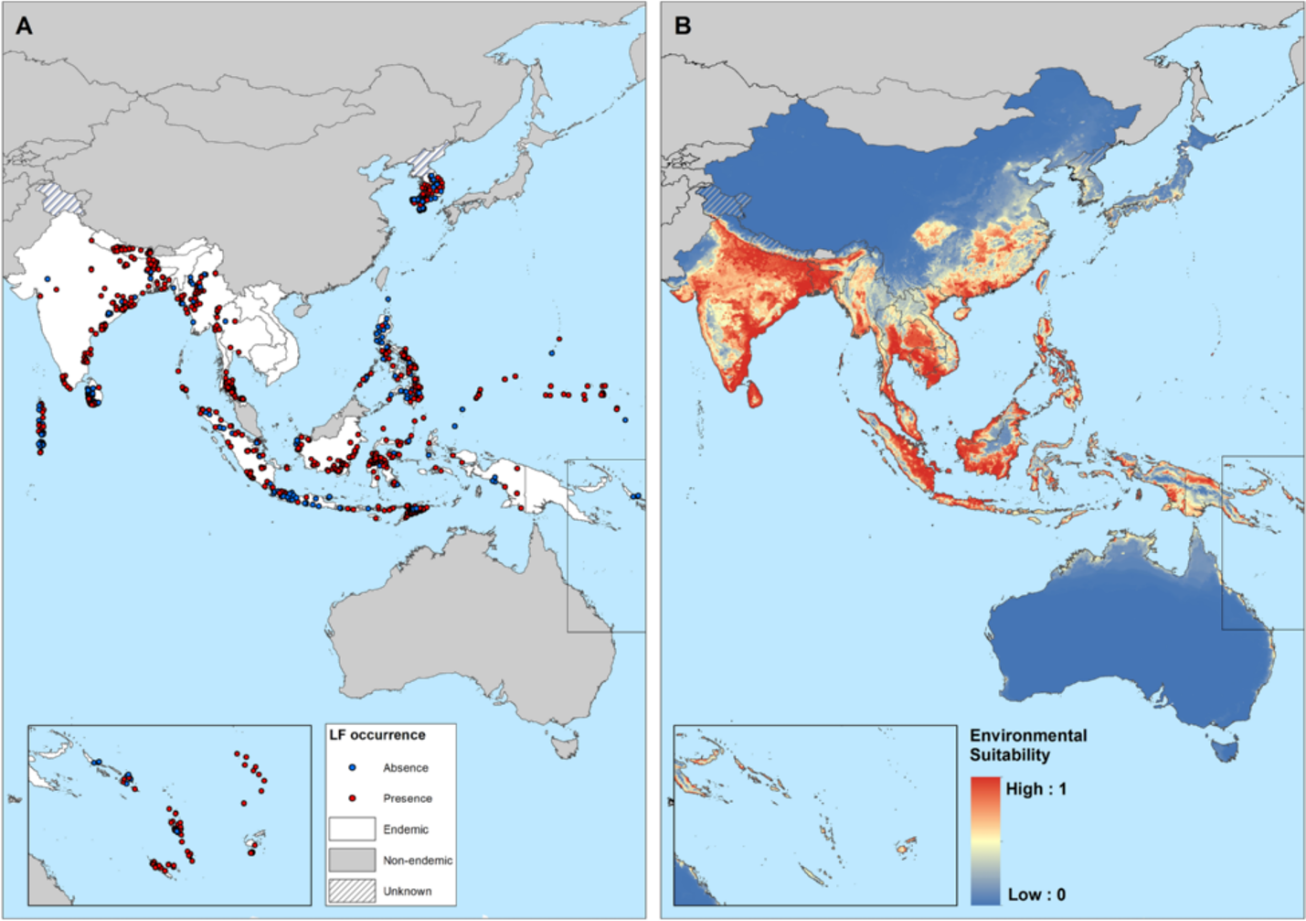
**Reported and predicted distribution of lymphatic filariasis in South-Asia and Western Pacific. (A)** Observed occurrence and absence of LF and **(B)** environmental suitability for lymphatic filariasis transmission, as predicted by the final boosted regression trees model, in South-east Asia and western Pacific.

Figures 7, 8 and 9 present the observed occurrence and absence of LF and environmental suitability for LF transmission for each region. In Africa, LF transmission is predicted to occur across much of coastal and savannah West Africa (Figure 7) but is restricted mainly to the coastal areas of east and southern Africa. The predicted distribution in central and southern Africa is uneven, with large foci in northeast of South Sudan (Upper Nile and Jonglei), Uganda, eastern Democratic Republic of Congo (Bas Congo, Bandundu and Equateur provinces), southeast Zambia and southern Malawi.

In the Americas region, LF transmission is predicted to occur throughout north and north-east regions of South-America, Central America, major islands in the Caribbean region (Haiti and the Dominican Republic) and marginally in coastal areas of southern United States (Figure 8). LF has been eliminated from 20 countries in the Americas region, and known current endemicity is restricted to Brazil, Guyana and the Hispaniola (Dominican Republic and Haiti) [99].

**Figure 10:**
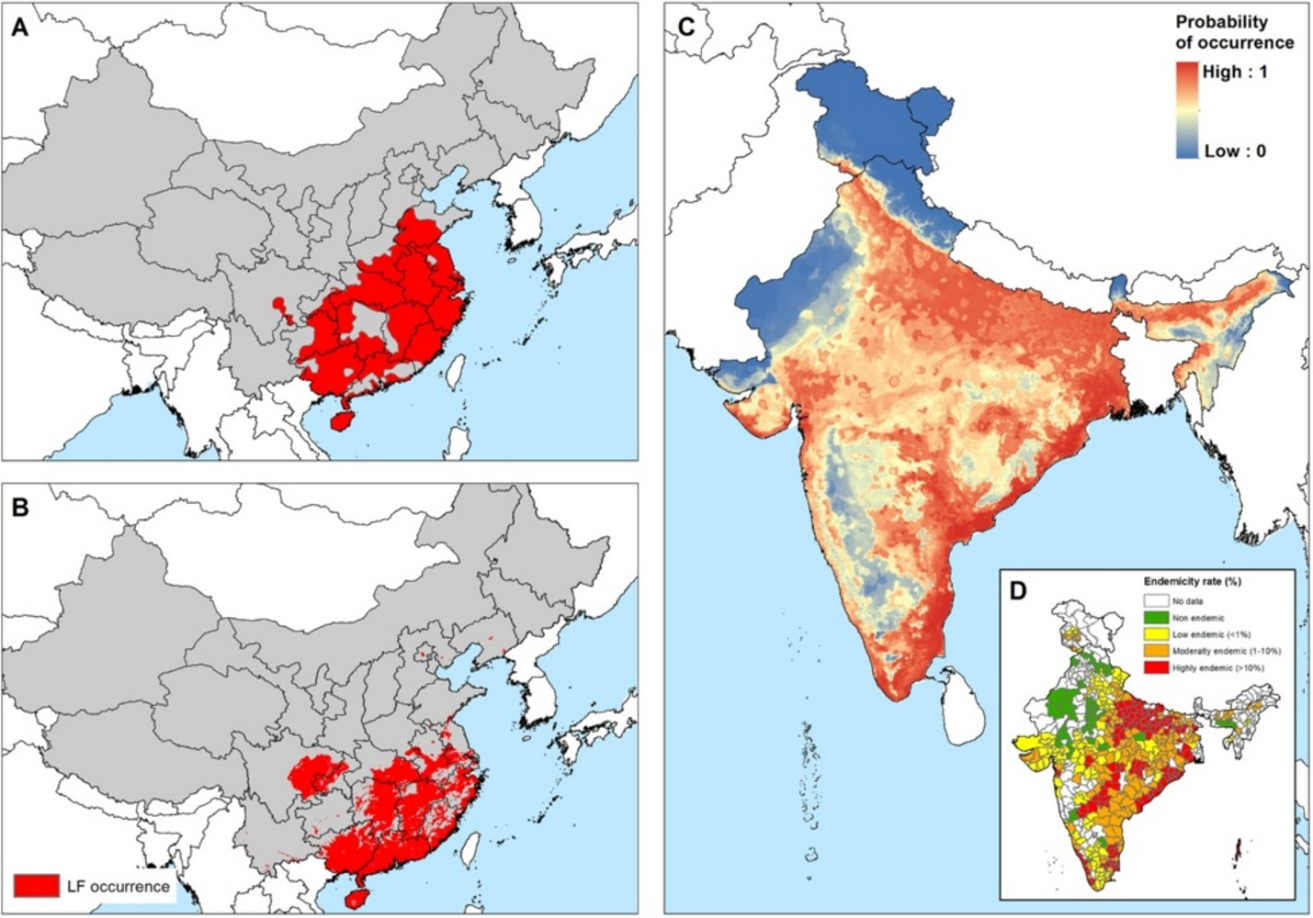
**Comparison of known historical distribution (A, D) and modelled distribution (B, C) in China and India. (A)** Historical distribution of LF in China (1950-1970) modified from Kimura *et al*. [20] and **(D)** district-level endemicity map in India based on historical data (prior to 2000) from Sabesan *et al*. [143].

In Asia and western Pacific, LF transmission is predicted to occur in the east of India, Sri Lanka, much of Southeast Asia and southeast China, Papua New Guinea, the northern coast of Australia and southern Japan (Figure 9). LF has been eliminated in China (2007), Japan (1980s) and South Korea (2008), but the predicted environmental suitability corresponds well with the known historical, pre-control distribution [11],[17],[20] (Figure 10).

## Discussion

Here we present a first global map of the distribution and transmission limits of LF. This work is opportune as it provides a basis for tracking and interpreting progress in control over time and can define the pre-control transmission limits, which, in turn, can inform the intensity and duration of control [2],[103]. Our work additionally provides insight to post-MDA surveillance by identifying areas of highest transmission which may be more prone to the resurgence of transmission following cessation of interventions.

In the current analysis we identify the environmental limits of potential transmission. We demonstrate that the probability of LF transmission increases with increasing precipitation, temperature and certain vegetation types but decreases with increasing altitude (Figure 4). These findings are consistent with previous analyses of environmental correlates at continental [49],[50] and country [52],[104],[105] scales and are, undoubtedly, linked to temperature-related variation in vector survival and parasite development within the vector [82],[84],[85],[106]. Our risk map, developed using boosted regression tree modelling, shows that the environmental conditions suitable for LF transmission occurs throughout the forest and savannah regions of West Africa, coastal east Africa and Madagascar and restricted foci in central and southern Africa. Suitable environmental conditions also occur across tropical areas of south and South-east Asia and the Pacific as well as large areas of Central and South America, including the southern States of America. Interestingly, however, active transmission in Central and South America is restricted to isolated foci; the possible reasons for this discrepancy are discussed below. Our predictions of environmental suitability in temperate regions are consistent with documented historical distributions prior to large scale intervention and local elimination in Japan, South Korea, and southern China [15]-[19] (Figure 10), the north coast of Australia [107] and southeastern coast of United States [99].

The probability of LF transmission is additionally associated with population density. Such an association probably reflects differences in the distribution of different vector species and their habitat preference and susceptibility to LF [108]-[110]. In rural areas of Africa, LF is transmitted by *Anopheles* species, whereas in urban settings in east Africa and the Nile Delta transmission is by *Culex quinquefasciatus*[111],[112]. In West Africa, *Culex* mosquitoes, although widely distributed [113], are considered refractory to infection [114]-[116], although some studies have demonstrated compatibility between West African strains of *W. bancrofti* and *Culex* and *Mansonia* mosquitoes [117]-[120]. *Culex* transmission also occurs in Asia [121]-[126] and *Culex quinquefasciatus* is the only vector known in the Americas [127],[128]. The true extent of LF transmission in urban settings, especially in sub-Saharan Africa, remains poorly documented [30],[129],[130], and further work is warranted.

Our work provides interesting insight into the regional distributions of LF. In sub-Saharan Africa, LF transmission is highly heterogeneous (Figure 7), with the highest potential risk in the forest and savannah regions of West Africa and coastal areas of eastern Africa and Madagascar. Scattered and relatively small areas of high-moderate risk are predicted in central Africa. This distribution of LF across Africa corresponds well with the known historical distribution of LF on the continent [17],[18] and predicts transmission in countries which have yet to be extensively mapped, such as Gabon [131] or Angola [132]. Our predictions indicate a more geographically restricted distribution of LF (Figure 7) than earlier spatial predictions using environmental factors [49],[50]. Our analysis included some 4,624 surveys across most endemic countries in Africa, whereas previous analysis [49],[50] included fewer than 700 surveys which were mainly concentrated in West Africa, Egypt, Sudan, Kenya, Tanzania and Madagascar; studies which were typically conducted in known areas of transmission. Such paucity and biased clustering of data coupled with the use of regression-based modelling will result in the smoothing of prevalence across large areas and overestimate prevalence in unsurveyed areas [133],[134]. Our use of BRT modelling overcomes the geographical bias of data by the use of pseudo absences [135], which are randomly generated from areas known to be unsuitable for mosquito breeding or non-endemic for LF.

Our risk map predicts widespread environmental suitability of LF in Central and South America, whereas active transmission is known to occur only in isolated foci [136]. We suggest two possible reasons for this discrepancy. First, historically, LF occurred across 20 countries and territories in the region [19],[99], including islands of the Caribbean and coastal areas of southeast Unites States, where indigenous cases were reported as far north as Philadelphia, until 1930s [99]. A combination of intensive vector control and improvements in public sanitary works resulted in the local elimination in these settings. Second, the transmission of LF in the Americas is strongly influenced by historical socioeconomic and demographic factors, rather than environmental factors. Transmission of LF was introduced to the Americas by slaves transported from West Africa to work in sugarcane plantations during the 17th and 18th centuries [137]. Initially, large numbers of slaves were brought to Barbados and Brazil, and subsequently sent to other islands of the Caribbean, to the north American colonies (South Carolina and Virginia) and northern countries of South America (Venezuela, Guyana, French Guiana, and Suriname) [17],[19],[99]. Upon introduction, *W. bancrofti* readily adapted from transmission by African *Anopheles* mosquitoes to transmission by *Culex* mosquitoes commonly found in the overcrowded and insanitary towns and cities of the Americas. However, it appears that transmission has remained restricted to those areas where the disease was firstly introduced. In settings where socioeconomic and sanitary conditions have improved and interventions have been implemented in recent decades, the disease has gradually disappeared, for example in the Caribbean region [138]. In those countries with active transmission today - Brazil, the Dominican Republic, Guyana and Haiti [39],[139], infection occurs mainly in urban settings and is strongly associated with poor socioeconomic conditions [140]-[142]. Future work will explore the interplay of environmental, socioeconomic factors and coverage of interventions on the distribution and prevalence of LF.

The distribution of LF in Asia exhibits marked regional trends. In India, high transmission occurs across the northern Indian (Gangetic) plain which borders Nepal and Bangladesh, eastern and south western coastal areas, areas in the central Deccan region in the south and on the Andaman and Nicobar Islands (Figure 9). Moderate-low transmission occurs in inland areas of southwestern India. Such a distribution is consistent with previous district-level mapping [143] (Figure 10D) and a previous national-level environmental risk model [104]. The predicted environmental limits for China, Japan and South Korea correspond well with the historical distribution prior to large control and local elimination [20],[144],[145] (Figure 10A-B). Although LF has been declared eliminated in China, the vectors remain and occasional imported cases have been reported [146], so that long-term surveillance is required to ensure that recrudescence does not occur. In contrast to the predictions for India and east Asia, our environmental-based map overestimates risk in the southeast Asian countries of Vietnam, Cambodia, Thailand and PDR Laos, which are considered to have limited transmission [147]. In Thailand, *B. malayi* transmission occurs in the south of the country [102] and *W. bancrofti* is endemic in the western provinces bordering Myanmar [148], but control measures implemented since the 1960s have dramatically reduced transmission [102].

We have sought to conduct an exhaustive search assembly of historical and contemporary data on LF occurrence and prevalence and have used rigorous methodology to predict the occurrence of LF transmission, but recognise a number of limitations. The BRT model presented here is driven by environmental parameters and spatial configuration of habitats that allow persistence of species in landscapes. This modelling approach has been applied successfully to map the distribution of mosquito-borne diseases, such as dengue and malaria, which are transmitted by one mosquito genera with limited species diversity within geographical regions [96],[149]. LF is unique among mosquito-borne diseases because it is transmitted by mosquito species belonging to five genera: *Aedes, Anopheles, Culex, Mansonia* and *Ochlerotatus*. In Africa, where LF is transmitted principally by *Anopheles* species, the disease distribution corresponds well with the known historical distribution patterns across the continent. This is not surprising because the environmental factors determining the abundance and distribution of *Anopheles* mosquitoes across Africa have not changed much in the peri-domestic environment in the past 20 years. However, our model performs less well in areas where LF is transmitted by *Culex* mosquitoes, especially *Culex quinquefasciatus*, which is predominately an urban mosquito with breeding habits that are influenced more by human activity than environmental factors [150],[151]. It follows, therefore, that environmental factors impact on transmission differently for different mosquito species. For example, precipitation, an important determinant in our environmental model, it likely to affect *Anopheles* and *Culex* species differently. While frequent rainfall generally tends to increase the densities of adult mosquitoes by producing breeding sites, their densities may be reduced by the flushing of sites when precipitation is high. It may be that anopheline and culicine mosquitoes differ in their response to heavy rainfall since they breed in different habitats. In areas suitable for transmission by *Culex quinquefasciatus*, human factors, such as sanitary and housing conditions, may play an important role in transmission. Our model may have therefore overestimated the current limits for LF in *Culex*-transmitted areas.

We additionally recognise the limitations of the current empirical evidence base, especially in regard to unpublished surveys that found an absence of transmission. We did not perform age correction of prevalence data since our current focus was on the occurrence of transmission. For similar reasons we did not adjust for differences in sensitivity of various diagnostic methods [152]. Future work will seek to model the prevalence of LF infection and will take into account such differences in age patterns and diagnostic method. Finally, our study focused on the environmental limits of LF transmission and, as discussed above, we recognise our model does not capture the socioeconomic and intervention-related dynamics of transmission. This is the subject of ongoing work investigating the spatiotemporal distribution of LF and the degree to which changes are related to the scaling-up of interventions or other factors.

## Conclusions

Despite the limitations and caveats acknowledged above, the assembled database represents a unique resource and the global map provides the best currently available indication of the global distribution of LF, past and present. Consistent with the open access approach of the Global Atlas of Helminth Infection, the assembled data and developed maps are publicly available (www.thiswormyworld.org). As the global LF community moves towards elimination, the assembled data maps and model predictions will help track progress and increase the cost-effectiveness of surveillance activities post-control.

## Endnotes

^a^AFRO/EMRO region: Seychelles and Mayotte; AMRO region: Antigua & Barbuda, Costa Rica, Cuba, French Guiana, Martinique, Puerto Rico, Saint Lucia, Saint Vincent and the Grenadines, Suriname, Trinidad & Tobago, Venezuela and Virgin Islands, United States of America; and WPRO region: Cook Islands, Palau and Solomon Islands.

## Authors’ information

Jorge Cano and Maria P Rebollo joint first authors.

## Additional file

## Electronic supplementary material

Additional file 1: Characteristics of surveys included in the lymphatic filariasis database (extended table).(PDF 86 KB)

## References

[1] Taylor MJ, Hoerauf A, Bockarie M (2010). Lymphatic filariasis and onchocerciasis. Lancet.

[2] Meeting of the International Task Force for Disease Eradication, January 2014. Wkly Epidemiol Rec. 2014, 89: 153-160. 24754045

[3] Kanjanopas K, Choochote W, Jitpakdi A, Suvannadabba S, Loymak S, Chungpivat S, Nithiuthai S (2001). Brugia malayi in a naturally infected cat from Narathiwat Province, southern Thailand. Southeast Asian J Trop Med Public Health.

[4] Lek-Uthai U, Tomoen W (2005). Susceptibility of Mansonia uniformis to Brugia malayi microfilariae from infected domestic cat. Southeast Asian J Trop Med Public Health.

[5] Ottesen EA, Duke BO, Karam M, Behbehani K (1997). Strategies and tools for the control/elimination of lymphatic filariasis. Bull World Health Organ.

[6] Preventive Chemotherapy in Human Helminthiasis. 2006, World Health Organization, Geneva

[7] Bockarie MJ, Molyneux DH (2009). The end of lymphatic filariasis?. BMJ.

[8] WHO position statement on integrated vector management to control malaria and lymphatic filariasis. Wkly Epidemiol Rec. 2011, 86: 121-127. 21438441

[9] Webber RH, Webber RH (1979). Eradication of Wuchereria bancrofti infection through vector control. Trans R Soc Trop Med Hyg.

[10] Ramzy RM, El Setouhy M, Helmy H, Ahmed ES, Abd Elaziz KM, Farid HA, Shannon WD, Weil GJ (2006). Effect of yearly mass drug administration with diethylcarbamazine and albendazole on bancroftian filariasis in Egypt: a comprehensive assessment. Lancet.

[11] Tada I (2011). Lymphatic filariasis and its control in Japan -the background of success. Trop Med Health.

[12] De-Jian S, Xu-Li D, Ji-Hui D (2013). The history of the elimination of lymphatic filariasis in China. Infect Dis Poverty.

[13] Sodahlon YK, Dorkenoo AM, Morgah K, Nabiliou K, Agbo K, Miller R, Datagni M, Seim A, Mathieu E (2013). A success story: Togo is moving toward becoming the first Sub-Saharan African nation to eliminate lymphatic filariasis through mass drug administration and countrywide morbidity alleviation. PLoS Negl Trop Dis.

[14] Hawking F (1957). The distribution of Bancroftian filariasis in Africa. Bull World Health Organ.

[15] Hawking F, Denham DA (1976). The distribution of human filariasis throughout the world. Part I. the Pacific Region, including New Guinea. Trop Dis Bull.

[16] Hawking F, Hawking F (1976). The distribution of human filariasis throughout the world. Part II. Asia. Trop Dis Bull.

[17] Sasa M (1976). Human Filariasis. A Global Survey of Epidemiology and Control.

[18] Hawking F (1977). The distribution of human filariasis throughout the world. Part III. Africa. Trop Dis Bull.

[19] Hawking F (1979). The distribution of human filariasis throughout the world part IV. America. Trop Dis Bull.

[20] Kimura E, Rim HJ, Dejian S, Weerasooriya M (2005). Filariasis in Asia and Western Pacific Islands.

[21] Brygoo ER (1958). La filariose humain a Madagascar. Arch Inst Pasteur Madagascar.

[22] OCCGE: Rapport final de la neuviem conference technique de I’OCCGE, 21-25 April 1969. Bobo-Dioulasso, Burkina Faso. 1969.

[23] Brengues J, Bouchité B, Nelson G, Ouedraogo C, Gbaguidi P, Dyemkouma A, Ochoumare J (1975). La filariose de Bancroft en Afrique de l’Ouest.

[24] Kuhlow F, Zielke E (1976). Distribution and prevalence of Wuchereria bancrofti in various parts of Liberia. Tropenmed Parasitol.

[25] Ou ZY, Luo JM, Luo XC (1991). The filariasis situation in Guangdong Province, China. Southeast Asian J Trop Med Public Health.

[26] Apiwathnasorn C, Kanjanopas K, Thammapalo S, Loymak S, Samung Y, Prummongkol S, Molyneux D (2003). Application of GIS to the characterization of filariasis transmission in Narathiwat Province. Southeast Asian J Trop Med Public Health.

[27] Michael E, Bundy DA, Grenfell BT (1996). Re-assessing the global prevalence and distribution of lymphatic filariasis. Parasitology.

[28] Hassan AN, Dister S, Beck L (1998). Spatial analysis of lymphatic filariasis distribution in the Nile Delta in relation to some environmental variables using geographic information system technology. J Egypt Soc Parasitol.

[29] Gyapong JO, Remme JH (2001). The use of grid sampling methodology for rapid assessment of the distribution of bancroftian filariasis. Trans R Soc Trop Med Hyg.

[30] Gyapong JO, Kyelem D, Kleinschmidt I, Agbo K, Ahouandogbo F, Gaba J, Owusu-Banahene G, Sanou S, Sodahlon YK, Biswas G, Kale OO, Molyneux DH, Roungou JB, Thomson MC, Remme J (2002). The use of spatial analysis in mapping the distribution of bancroftian filariasis in four West African countries. Ann Trop Med Parasitol.

[31] Mukoko DA, Pedersen EM, Masese NN, Estambale BB, Ouma JH (2004). Bancroftian filariasis in 12 villages in Kwale district, Coast province, Kenya - variation in clinical and parasitological patterns. Ann Trop Med Parasitol.

[32] Boyd A, Won KY, McClintock SK, Donovan CV, Laney SJ, Williams SA, Pilotte N, Streit TG, de Rochars MV B, Lammie PJ (2010). A community-based study of factors associated with continuing transmission of lymphatic filariasis in Leogane, Haiti. PLoS Negl Trop Dis.

[33] Weil GJ, Lammie PJ, Weiss N (1997). The ICT filariasis test: a rapid-format antigen test for diagnosis of bancroftian filariasis. Parasitol Today.

[34] Bhumiratana A, Koyadun S, Suvannadabba S, Karnjanopas K, Rojanapremsuk J, Buddhirakkul P, Tantiwattanasup W (1999). Field trial of the ICT filariasis for diagnosis of Wuchereria bancrofti infections in an endemic population of Thailand. Southeast Asian J Trop Med Public Health.

[35] Phantana S, Sensathein S, Songtrus J, Klagrathoke S, Phongnin K (1999). ICT filariasis test: a new screening test for Bancroftian filariasis. Southeast Asian J Trop Med Public Health.

[36] Rahmah N, Taniawati S, Shenoy RK, Lim BH, Kumaraswami V, Anuar AK, Hakim SL, Hayati MI, Chan BT, Suharni M, Ramachandran CP (2001). Specificity and sensitivity of a rapid dipstick test (Brugia Rapid) in the detection of Brugia malayi infection. Trans R Soc Trop Med Hyg.

[37] Supali T, Rahmah N, Djuardi Y, Sartono E, Rückert P, Fischer P (2004). Detection of filaria-specific IgG4 antibodies using Brugia Rapid test in individuals from an area highly endemic for Brugia timori. Acta Trop.

[38] Melrose W, Rahmah N (2006). Use of Brugia Rapid dipstick and ICT test to map distribution of lymphatic filariasis in the Democratic Republic of Timor-Leste. Southeast Asian J Trop Med Public Health.

[39] Sustaining the drive to overcome the global impact of neglected tropical diseases: second WHO report on neglected diseases. 2013, World Health Organization, Geneva

[40] Sherchand JB, Obsomer V, Thakur GD, Hommel M (2003). Mapping of lymphatic filariasis in Nepal. Filaria J.

[41] de Rochars MV B, Milord MD, St Jean Y, Desormeaux AM, Dorvil JJ, Lafontant JG, Addiss DG, Streit TG (2004). Geographic distribution of lymphatic filariasis in Haiti. Am J Trop Med Hyg.

[42] Ngwira BM, Tambala P, Perez AM, Bowie C, Molyneux DH (2007). The geographical distribution of lymphatic filariasis infection in Malawi. Filaria J.

[43] Koroma JB, Bangura MM, Hodges MH, Bah MS, Zhang Y, Bockarie MJ (2012). Lymphatic filariasis mapping by immunochromatographic test cards and baseline microfilaria survey prior to mass drug administration in Sierra Leone. Parasit Vectors.

[44] Okorie PN, Ademowo GO, Saka Y, Davies E, Okoronkwo C, Bockarie MJ, Molyneux DH, Kelly-Hope LA (2013). Lymphatic filariasis in Nigeria; Micro-stratification Overlap Mapping (MOM) as a prerequisite for cost-effective resource utilization in control and surveillance. PLoS Negl Trop Dis.

[45] Shiferaw W, Kebede T, Graves PM, Golasa L, Gebre T, Mosher AW, Tadesse A, Sime H, Lambiyo T, Panicker KN, Richards FO, Hailu A (2012). Lymphatic filariasis in western Ethiopia with special emphasis on prevalence of Wuchereria bancrofti antigenaemia in and around onchocerciasis endemic areas. Trans R Soc Trop Med Hyg.

[46] Wijegunawardana NDD, Gunawardene YINS, Manamperi A, Senarathne H, Abeyewickreme W (2012). Geographic information system (GIS) mapping of lymphatic filariasis endemic areas of Gampaha District, Sri Lanka based on epidemiological and entomological screening. Southeast Asian J Trop Med Public Health.

[47] Southgate BA (1992). Intensity and efficiency of transmission and the development of microfilaraemia and disease: their relationship in lymphatic filariasis. J Tropical Med Hygiene.

[48] Brooker S, Michael E (2000). The potential of geographical information systems and remote sensing in the epidemiology and control of human helminth infections. Adv Parasitol.

[49] Lindsay SW, Thomas CJ (2000). Mapping and estimating the population at risk from lymphatic filariasis in Africa. Trans R Soc Trop Med Hyg.

[50] Slater H, Michael E (2012). Predicting the current and future potential distributions of lymphatic filariasis in Africa using maximum entropy ecological niche modelling. PLoS One.

[51] Slater H, Michael E (2013). Mapping, Bayesian geostatistical analysis and spatial prediction of lymphatic filariasis prevalence in Africa. PLoS One.

[52] Stanton MC, Molyneux DH, Kyelem D, Bougma RW, Koudou BG, Kelly-Hope LA (2013). Baseline drivers of lymphatic filariasis in Burkina Faso. Geospat Health.

[53] Hassan AN (2004). Bancroftian filariasis: spatial patterns, environmental correlates and landscape predictors of disease risk. J Egypt Soc Parasitol.

[54] Sabesan S, Raju HK, Srividya A, Das PK (2006). Delimitation of lymphatic filariasis transmission risk areas: a geo-environmental approach. Filaria J.

[55] Michael E, Malecela-Lazaro MN, Simonsen PE, Pedersen EM, Barker G, Kumar A, Kazura JW (2004). Mathematical modelling and the control of lymphatic filariasis. Lancet Infect Dis.

[56] Garrett-Jones C (1964). Prognosis for interruption of malaria transmission through assessment of the mosquito’s vectorial capacity. Nature.

[57] Duerr HP, Dietz K, Eichner M (2005). Determinants of the eradicability of filarial infections: a conceptual approach. Trends Parasitol.

[58] Poncon N, Tran A, Toty C, Luty AJ, Fontenille D (2008). A quantitative risk assessment approach for mosquito-borne diseases: malaria re-emergence in southern France. Malar J.

[59] Gambhir M, Michael E (2008). Complex ecological dynamics and eradicability of the vector borne macroparasitic disease, lymphatic filariasis. PLoS One.

[60] Brooker S, Hotez PJ, Bundy DA (2010). The global atlas of helminth infection: mapping the way forward in neglected tropical disease control. PLoS Negl Trop Dis.

[61] Michael E, Malecela MN, Zervos M, Kazura JW (2008). Global eradication of lymphatic filariasis: the value of chronic disease control in parasite elimination programmes. PLoS One.

[62] Eberhard ML, Lammie PJ (1991). Laboratory diagnosis of filariasis. Clin Lab Med.

[63] Research on Rapid Geographical Assessment of Bancroftian Filariasis. 1998, World Health Organization, Geneva

[64] Srividya A, Lall R, Ramaiah KD, Ramu K, Hoti SL, Pani SP, Das PK (2000). Development of rapid assessment procedures for the delimitation of lymphatic filariasis-endemic areas. Trop Med Int Health.

[65] Chu BK, Deming M, Biritwum NK, Bougma WR, Dorkenoo AM, El-Setouhy M, Fischer PU, Gass K, Gonzalez de Pena M, Mercado-Hernandez L, Kyelem D, Lammie PJ, Flueckiger RM, Mwingira UJ, Noordin R, Offei Owusu I, Ottesen EA, Pavluck A, Pilotte N, Rao RU, Samarasekera D, Schmaedick MA, Settinayake S, Simonsen PE, Supali T, Taleo F, Torres M, Weil GJ, Won KY (2013). Transmission assessment surveys (TAS) to define endpoints for lymphatic filariasis mass drug administration: a multicenter evaluation. PLoS Negl Trop Dis.

[66] King JD, Eigege A, Umaru J, Jip N, Miri E, Jiya J, Alphonsus KM, Sambo Y, Graves P, Richards F (2012). Evidence for stopping mass drug administration for lymphatic filariasis in some, but not all local government areas of plateau and nasarawa States, Nigeria. Am J Trop Med Hyg.

[67] Report on the mid-Term Assessment of Microfilaraemia Reduction in Sentinel Sites of 13 Countries of the Global Programme to Eliminate Lymphatic Filariasis. Weekly Epidemiological Record. 2004, 357-368. 357-368, 79 15631012

[68] Msyamboza K, Ngwira B, Banda R, Mkwanda S, Brabin B (2010). Sentinel surveillance of lymphatic filariasis, schistosomiasis soil transmitted helminths and malaria in rural southern Malawi. Malawi Med J.

[69] Solomon AW, Engels D, Bailey RL, Blake IM, Brooker S, Chen JX, Chen JH, Churcher TS, Drakeley CJ, Edwards T, Fenwick A, French M, Gabrielli AF, Grassl NC, Harding-Esch EM, Holland MJ, Koukounari A, Lammie PJ, Leslie J, Mabey DC, Rhajaoui M, Secor WE, Stothard JR, Wei H, Willingham AL, Zhou XN, Peeling RW (2012). A diagnostics platform for the integrated mapping, monitoring, and surveillance of neglected tropical diseases: rationale and target product profiles. PLoS Negl Trop Dis.

[70] Stoll NR (1947). This wormy world. J Parasitol.

[71] Guerra CA, Hay SI, Lucioparedes LS, Gikandi PW, Tatem AJ, Noor AM, Snow RW (2007). Assembling a global database of malaria parasite prevalence for the Malaria Atlas Project. Malar J.

[72] Hay SI, Guerra CA, Gething PW, Patil AP, Tatem AJ, Noor AM, Kabaria CW, Manh BH, Elyazar IR, Brooker S, Smith DL, Moyeed RA, Snow RW (2009). A world malaria map: plasmodium falciparum endemicity in 2007. PLoS Med.

[73] Brooker S, Rowlands M, Haller L, Savioli L, Bundy DA (2000). Towards an atlas of human helminth infection in sub-Saharan Africa: the use of geographical information systems (GIS). Parasitol Today.

[74] Brooker S, Kabatereine NB, Smith JL, Mupfasoni D, Mwanje MT, Ndayishimiye O, Lwambo NJ, Mbotha D, Karanja P, Mwandawiro C, Muchiri E, Clements AC, Bundy DA, Snow RW (2009). An updated atlas of human helminth infections: the example of East Africa. Int J Health Geogr.

[75] Hurlimann E, Schur N, Boutsika K, Stensgaard AS, Laserna de Himpsl M, Ziegelbauer K, Laizer N, Camenzind L, Di Pasquale A, Ekpo UF, Simoonga C, Mushinge G, Saarnak CF, Utzinger J, Kristensen TK, Vounatsou P (2011). Toward an open-access global database for mapping, control, and surveillance of neglected tropical diseases. PLoS Negl Trop Dis.

[76] *The Second Administrative Level Boundaries data set project.*, [http://www.unsalb.org/]

[77] *GADM database of Global Administrative Areas.*, [http://www.gadm.org/]

[78] *Bing Maps.*, [http://www.bing.com/maps/]

[79] *NGA-GEOnet Names Server (Country Files).*, [http://earth-info.nga.mil/gns/html/index.html]

[80] *Fuzzy Gazetteer.*, [http://isodp.hof-university.de/fuzzyg/query/]

[81] *Open Street Map.*, [http://www.openstreetmap.org/]

[82] Lindsay SW, Denham DA, McGreevy PB (1984). The effect of humidity on the transmission of Brugia pahangi infective larvae to mammalian hosts by Aedes aegypti. Trans R Soc Trop Med Hyg.

[83] Samarawickrema WA, Spears GF, Sone F, Ichimori K, Cummings RF (1985). Filariasis transmission in Samoa. II. Some factors related to the development of microfilariae in the intermediate host. Ann Trop Med Parasitol.

[84] Lardeux F, Cheffort J (1997). Temperature thresholds and statistical modelling of larval Wuchereria bancrofti (Filariidea:Onchocercidae) developmental rates. Parasitology.

[85] Lardeux F, Cheffort J (2001). Ambient temperature effects on the extrinsic incubation period of Wuchereria bancrofti in Aedes polynesiensis: implications for filariasis transmission dynamics and distribution in French Polynesia. Med Vet Entomol.

[86] de Souza D, Kelly-Hope L, Lawson B, Wilson M, Boakye D (2010). Environmental factors associated with the distribution of Anopheles gambiae s.s in Ghana; an important vector of lymphatic filariasis and malaria. PLoS One.

[87] *Global Climate data.*, [http://www.worldclim.org/]

[88] *Consortium for Spatial Information.*, [http://www.cgiar-csi.org/]

[89] *IRI/LDEO Climate Library.*, [http://iridl.ldeo.columbia.edu/]

[90] *Global Land Cover Map.*, [http://due.esrin.esa.int/globcover/]

[91] *Gridded Population of the World (GPW), v3.*, [http://sedac.ciesin.columbia.edu/data/collection/gpw-v3]

[92] Pullan RL, Brooker SJ (2012). The global limits and population at risk of soil-transmitted helminth infections in 2010. Parasit Vectors.

[93] *Travel Time to Major Cities: A Global map of Accessibility.*], [http://bioval.jrc.ec.europa.eu/products/gam/index.htm]

[94] Elith JH, Graham CP, Anderson R, Dudík M, Ferrier S, Guisan AJ, Hijmans R, Huettmann FR, Leathwick J, Lehmann A, Li J, Lohmann LG, Loiselle BA, Manion G, Moritz C, Nakamura M, Nakazawa Y, Overton JMC, Townsend Peterson AT, Phillips SJ, Richardson K, Scachetti-Pereira R, Schapire RE, Soberón J, Williams S, Wisz MS, Zimmermann NE (2006). Novel methods improve prediction of species’ distributions from occurrence data. Ecography.

[95] Bhatt S, Gething PW, Brady OJ, Messina JP, Farlow AW, Moyes CL, Drake JM, Brownstein JS, Hoen AG, Sankoh O, Myers MF, George DB, Jaenisch T, Wint GR, Simmons CP, Scott TW, Farrar JJ, Hay SI (2013). The global distribution and burden of dengue. Nature.

[96] Sinka ME, Bangs MJ, Manguin S, Rubio-Palis Y, Chareonviriyaphap T, Coetzee M, Mbogo CM, Hemingway J, Patil AP, Temperley WH, Gething PW, Kabaria CW, Burkot TR, Harbach RE, Hay SI (2012). A global map of dominant malaria vectors. Parasit Vectors.

[97] De’ath G (2007). Boosted trees for ecological modeling and predictions. Ecology.

[98] Elith J, Leathwick JR, Hastie T (2008). A working guide to boosted regression trees. J Anim Ecol.

[99] Addiss D, Chuke S (2002). Lymphatic Filariasis in the Americas: An Epidemiologic History. Centers for Disease Control and Prevention.

[100] Organization WH (2003). Global Programme to Eliminate Lymphatic Filariasis. Annual Report on Lymphtic Filariasis 2005.

[101] Ninth Workshop for Pacific Lymphatic Filariasis Programme Managers. WHO Meeting report, (WP)MVP/PIC/CPC/12/001-E. 2007, vol. RS/2007/GE/20(FIJ)

[102] Iyengar MO (1953). Filariasis in Thailand. Bull World Health Organ.

[103] Rebollo MP, Bockarie MJ (2013). Toward the elimination of lymphatic filariasis by 2020: treatment update and impact assessment for the endgame. Expert Rev Anti-Infect Ther.

[104] Sabesan S, Raju HK, Subramanian S, Srivastava PK, Jambulingam P (2013). Lymphatic filariasis transmission risk map of india. Based on a geo-environmental risk model. Vector Borne Zoonotic Dis.

[105] Upadhyayula SM, Mutheneni SR, Kumaraswamy S, Kadiri MR, Pabbisetty SK, Yellepeddi VS (2012). Filaria monitoring visualization system: a geographical information system-based application to manage lymphatic filariasis in Andhra Pradesh, India. Vector Borne Zoonotic Dis.

[106] Rao SS, Iyengar MOT (1930). Studies on the influence of season on the development of Filaria bancrofti in Culex fatigans. Indian J Med Res.

[107] Boreham PFL, Marks EN (1986). Human filariasis in Australia: introduction, investigation and elimination. Proc R Soc Qld.

[108] Obiamiwe BA (1977). The influence of the gene sb in Culex pipiens on the development of sub-periodic Brugia malayi and Wuchereria bancrofti. Ann Trop Med Parasitol.

[109] Pothikasikorn J, Bangs MJ, Boonplueang R, Chareonviriyaphap T (2008). Susceptibility of various mosquitoes of Thailand to nocturnal subperiodic Wuchereria bancrofti. J Vector Ecol.

[110] Saeung A, Hempolchom C, Baimai V, Thongsahuan S, Taai K, Jariyapan N, Chaithong U, Choochote W (2013). Susceptibility of eight species members in the Anopheles hyrcanus group to nocturnally subperiodic Brugia malayi. Parasit Vectors.

[111] Pedersen EM, Kilama WL, Swai AB, Kihamia CM, Rwiza H, Kisumku UM (1999). Bancroftian filariasis on Pemba Island, Zanzibar, Tanzania: an update on the status in urban and semi-urban communities. Trop Med Int Health.

[112] Maxwell CA, Curtis CF, Haji H, Kisumku S, Thalib AI, Yahya SA (1990). Control of Bancroftian filariasis by integrating therapy with vector control using polystyrene beads in wet pit latrines. Trans R Soc Trop Med Hyg.

[113] Knight KL, Stone A (1977). A Catalog of the Mosquitoes of the World (Diptera: Culicidae).

[114] Subra R, Mouchet J (1967). Culex pipens fatigans Wiedemann in West Africa and its possible role in the transmission of Bancroft’s filariasis. WHO Mimeograph Document.

[115] Zielke E, Kuhlow F (1977). On the inheritance of susceptibility for infection with Wuchereria bancrofti in Culex pipiens fatigans. Tropenmed Parasitol.

[116] Jayasekera N, Curtis CF, Zielke E, Kuhlow F, Jansen CG, Chelliah RV (1980). The susceptibility of Liberian Culex quinquefasciatus to Wuchereria bancrofti in Sri Lanka. Tropenmed Parasitol.

[117] Udonsi JK (1988). Bancroftian filariasis in the Igwun Basin, Nigeria. An epidemiological, parasitological, and clinical study in relation to the transmission dynamics. Acta Trop.

[118] Anosike JC, Onwuliri CO (1992). Experimental Wuchereria bancrofti infection of Culex quinquefasciatus and Aedes aegypti. Angew Parasitol.

[119] Anosike JC, Nwoke BE, Ajayi EG, Onwuliri CO, Okoro OU, Oku EE, Asor JE, Amajuoyi OU, Ikpeama CA, Ogbusu FI, Meribe CO (2005). Lymphatic filariasis among the Ezza people of Ebonyi State, Eastern Nigeria. Ann Agric Environ Med.

[120] Ughasi J, Bekard HE, Coulibaly M, Adabie-Gomez D, Gyapong J, Appawu M, Wilson MD, Boakye DA (2012). Mansonia africana and Mansonia uniformis are vectors in the transmission of Wuchereria bancrofti lymphatic filariasis in Ghana. Parasit Vectors.

[121] Joe LK, Chow CY, Winoto RM, Rusad S, Rusad M (1958). [Filariasis in Djakarta, Indonesia]. Am J Trop Med Hyg.

[122] Surendran K, Pani SP, Soudarssanane MB, Srinivasa DK, Bordolai PC, Subramanian S (1996). Natural history, trend of prevalence and spectrum of manifestations of Bancroftian filarial disease in Pondicherry (South India). Acta Trop.

[123] Arunachalam N, Mariappan T, Vijayakumar KN, Sabesan S, Panicker KN (1996). Mattancherry urban agglomeration, a diminishing focus of lymphatic filariasis in Kerala. J Commun Dis.

[124] Jr TJD, Bhattacharya NC (1976). Clinical manifestations of Bancroftian filariasis in a suburb of Calcutta, India. Am J Trop Med Hyg.

[125] Oemijati S, Desowitz RS, Partono F, Pant CP, Mechfudin H, Sajidiman H (1975). Studies on filariasis in the Pacific. 4. The application of the membrane filter concentration technique to a survey of Wuchereria bancrofti filariasis in Kepu district, Jakarta, Indonesia. Southeast Asian J Trop Med Public Health.

[126] Oemijati S (1993). Current status of filariasis in Indonesia. Southeast Asian J Trop Med Public Health.

[127] Bonfim C, Lessa F, Oliveira C, Evangelista MJ, do Espirito Santo M, Meireles E, Pereira JC, Medeiros Z (2003). [The occurrence and distribution of lymphatic filariasis in Greater Metropolitan Recife: the case of an endemic area in Jaboatao dos Guararapes, Pernambuco, Brazil]. Cad Saude Publica.

[128] Braga C, Dourado I, Ximenes R, Miranda J, Alexander N (2005). Bancroftian filariasis in an endemic area of Brazil: differences between genders during puberty. Rev Soc Bras Med Trop.

[129] Terranella A, Eigiege A, Gontor I, Dagwa P, Damishi S, Miri E, Blackburn B, McFarland D, Zingeser J, Jinadu MY, Richards FO (2006). Urban lymphatic filariasis in central Nigeria. Ann Trop Med Parasitol.

[130] Gbakima AA, Appawu MA, Dadzie S, Karikari C, Sackey SO, Baffoe-Wilmot A, Gyapong J, Scott AL (2005). Lymphatic filariasis in Ghana: establishing the potential for an urban cycle of transmission. Trop Med Int Health.

[131] Richard-Lenoble D, Kombila M, Carme B, Gilles JC, Delattre PY (1980). [Prevalence of human filariasis with microfilaremia in Gabon]. Bull Soc Pathol Exot Filiales.

[132] Casaca VM (1966). [Contribution to the study of bancrofti filariasis in Angola. 1. Possibilities of its existence and probable distribution. 2. Bibliographic review and personal observations. An Inst Med Trop (Lisb).

[133] Reddy S, Dávalos LM (2003). Geographical sampling bias and its implications for conservation priorities in Africa. J Biogeography.

[134] Kadmon R, Farber O, Danin A (2004). Effect of roadside bias on the accuracy of predictive maps produced by bioclimatic models. Ecol Appl.

[135] Phillips SJ, Dudik M, Elith J, Graham CH, Lehmann A, Leathwick J, Ferrier S (2009). Sample selection bias and presence-only distribution models: implications for background and pseudo-absence data. Ecol Appl.

[136] Schneider MC, Aguilera XP, Barbosa da Silva J, Ault SK, Najera P, Martinez J, Requejo R, Nicholls RS, Yadon Z, Silva JC, Leanes LF, Periago MR (2011). Elimination of neglected diseases in Latin America and the Caribbean: a mapping of selected diseases. PLoS Negl Trop Dis.

[137] Laurence BR (1989). The global dispersal of Bancroftian filariasis. Parasitol Today.

[138] Rawlins SC, Lammie P, Tiwari T, Pons P, Chadee DD, Oostburg BF, Baboolal S (2000). Lymphatic filariasis in the Caribbean region: the opportunity for its elimination and certification. Rev Panam Salud Publica.

[139] Lymphatic Filariasis Elimination in the Americas: 12th Regional Program Managers Meeting. 2014, The Dominican Republic: Pan American Health Organization, Santo Domingo

[140] Raccurt CP, Lowrie RC, Katz SP, Duverseau YT (1988). Epidemiology of Wuchereria bancrofti in Leogane, Haiti. Trans R Soc Trop Med Hyg.

[141] Lymphatic Filariasis Elimination in the Americas: First Regional Program Managers Meeting. 2000, The Dominican Republic: Pan American Health Organization, Santo Domingo

[142] Bonfim C, Netto MJ, Pedroza D, Portugal JL, Medeiros Z (2009). A socioenvironmental composite index as a tool for identifying urban areas at risk of lymphatic filariasis. Trop Med Int Health.

[143] Sabesan S, Palaniyandi M, Das PK, Michael E (2000). Mapping of lymphatic filariasis in India. Ann Trop Med Parasitol.

[144] Global programme to eliminate lymphatic filariasis. Wkly Epidemiol Rec. 2008, 83: 333-348. 18788146

[145] Kimura E, Itoh M (2011). Filariasis in Japan some 25 years after its eradication. Trop Med Health.

[146] Zhang XC, Huang SY, Deng ZH, Ou ZY, Wu WP, Luo XC, Chen XX, Zhang QM, Lin RX, Ruan CW, Wang JL, Cui HE (2008). [Follow-up survey on the imported cases of lymphatic filariasis in Guangdong Province]. Zhongguo Ji Sheng Chong Xue Yu Ji Sheng Chong Bing Za Zhi.

[147] Sudomo M, Chayabejara S, Duong S, Hernandez L, Wu WP, Bergquist R (2010). Elimination of lymphatic filariasis in Southeast Asia. Adv Parasitol.

[148] Nuchprayoon S, Sanprasert V, Porksakorn C, Nuchprayoon I (2003). Prevalence of bancroftian filariasis on the Thai-Myanmar border. Asian Pac J Allergy Immunol.

[149] Brady OJ, Gething PW, Bhatt S, Messina JP, Brownstein JS, Hoen AG, Moyes CL, Farlow AW, Scott TW, Hay SI (2012). Refining the global spatial limits of dengue virus transmission by evidence-based consensus. PLoS Negl Trop Dis.

[150] Knudsen AB, Slooff R (1992). Vector-borne disease problems in rapid urbanization: new approaches to vector control. Bull World Health Organ.

[151] Adeleke MA, Mafiana CF, Idowu AB, Adekunle MF, Sam-Wobo SO (2008). Mosquito larval habitats and public health implications in Abeokuta, Ogun State, Nigeria. Tanzan J Health Res.

[152] McCarthy JS, Lustigman S, Yang G-J, Barakat RM, García HH, Sripa B, Willingham AL, Prichard RK, Basáñez M-G (2012). A research agenda for helminth diseases of humans: diagnostics for control and elimination programmes. PLoS Negl Trop Dis.

