# Supplementary material for: The global distribution and transmission limits of lymphatic filariasis: past and present
Source: Parasit Vectors. 2014 Oct 11;7:466. doi: 10.1186/s13071-014-0466-x (PMC4197264; doi:10.1186/s13071-014-0466-x)
Supplement: Supplementary file 1 — Additional file 1: Characteristics of surveys included in the lymphatic filariasis database (extended table).(PDF 86 KB) [file 13071_2014_466_MOESM1_ESM.pdf]

Additional file 2. Characteristics of surveys included in the lymphatic filariasis database (extended).

**WHO - regions**[&]	**AFRO/EMRO** N (%)	**SEARO** N (%)	**WPRO** N (%)	**AMRO** N (%)	**Total** N (%)
Current endemic countries	37	9	22	4	72
Countries with data	35	9	25	16	85
Number of surveys identified	5,468	1,764	1,322	479	9,033
Non-community data	673 (12.3)	246 (13.9)	201 (15.2)	43 (9.0)	1,163 (12.9)
**Geopositioned**	4,624 (84.6)	1,320 (74.8)	1058 (80.0)	418 (87.3)	**7,420 (82.1)**
Not geopositioned	171 (3.1)	198 (11.2)	63 (4.8)	18 (3.8)	432 (5.0)
Indicators concerning to community-level geopositioned surveys					
Geo-reliability					
1 (highly reliable)	3,829 (82.8)	936 (70.9)	859 (81.2)	387 (92.6)	6,011 (81.0)
2 (fairly reliable)	513 (11.1)	214 (16.2)	159 (15.0)	31 (7.4)	917 (12.4)
3 (less reliable)	155 (3.4)	109 (8.3)	32 (3.0)	-	296 (4.0)
4 (low reliable)	127 (2.7)	61 (4.6)	8 (0.8)	-	196 (2.6)
Surveys by period					
before 1990	1,157 (25.0)	688 (52.1)	774 (73.2)	316 (75.6)	2,935 (39.6)
1990-2000	441 (9.5)	146 (11.1)	116 (11.0)	70 (16.7)	773 (10.4)
2000-2010	2,040 (44.1)	486 (36.8)	159 (15.0)	32 (7.7)	2,717 (36.6)
2010-now	937 (20.3)	-	9 (0.9)	-	946 (12.7)
Unknown	49 (1.1)	-	-	-	49 (0.7)
Type of survey					
Mapping/prevalence	3,875 (83.8)	924 (70.0)	954 (90.2)	400 (95.7)	6,153 (82.9)
SS/Spot check	749 (16.2)	280 (21.2)	94 (8.9)	18 (4.3)	1,141 (15.4)
TAS	-	6 (0.5)	10 (0.9)	-	16 (0.2)
Passive recording*	-	110 (8.3)	-	-	110 (1.5)
Diagnostic method					
Clinical	24 (0.5)	119 (9.0)	5 (0.5)	1 (0.2)	149 (2.0)
Parasitological	1,939 (41.9)	1,086 (82.3)	892 (84.3)	370 (88.5)	4,287 (57.8)
Serological	2,423 (52.4)	69 (5.2.)	114 (10.8)	30 (7.2)	2,636 (35.5)
Other	238 (5.1)	46 (3.5)	47 (4.4)	17 (4.1)	348 (4.7)
MDA Implemented					
Unknown	19 (0.6)	36 (2.7)	-	18 (4.3)	84 (1.1)
Pre-intervention	3,918 (84.7)	827 (62.7)	558 (52.7)	312 (74.6)	5,615 (75.7)
Post-intervention	676 (14.6)	457 (34.6)	500 (47.3)	88 (21.1)	1,721 (23.2)
People surveyed (x1,000)					
Parasitological	979.73 (74.2)	1,994.09 (98.4)	449.44 (86.7)	14,161.1 (99.9)	17,584 (97.4)
ICT/Brugia test	263.58 (20.0)	12.98 (0.6)	52.23 (10.1)	8.07 (0.1)	337 (1.9)
Other	77.51 (5.9)	18.53 (0.9)	16.68 (3.2)	11.97 (0.1)	125 (0.7)

Total	1,320.8	2,025.6	518.4	14,181.1	18,045.9
Age ranges examined					
Unknown	1,051 (22.7)	47 (3.6)	27 (2.6)	5 (1.2)	1,130 (15.2)
≤ 15 years	115 (2.5)	77 (5.8)	27 (2.6)	40 (9.6)	259 (3.5)
16+ years	1,782 (38.5)	21 (1.6)	83 (7.8)	35 (8.4)	1,921 (25.9)
All ages	1,676 (36.2)	1,175 (89.0)	921 (87.1)	338 (89.0)	4,110 (55.4)
Morbidity data provided					
Hydrocele	1,018 (22.0)	30 (2.3)	46 (4.3)	1 (0.2)	1,095 (14.8)
Lymphoedema	1,004 (21.7)	230 (17.4)	97 (9.2)	13 (3.1)	1,344 (18.1)
Any clinical	382 (8.3)	221 (16.7)	34 (3.2)	4 (1.0)	641 (8.6)

[$] Include country reports, GAELF reports and 'grey' literature (unpublished reports)
[&]AFRO - African Regional Office, AMRO - Americas Regional Office, EMRO - Eastern Mediterranean Regional Office, SEARO - South East Asian Regional Office, WPRO - Western Pacific Regional Office
